# Systematic review of statistical approaches to quantify, or correct for, measurement error in a continuous exposure in nutritional epidemiology

**DOI:** 10.1186/s12874-017-0421-6

**Published:** 2017-09-19

**Authors:** Derrick A. Bennett, Denise Landry, Julian Little, Cosetta Minelli

**Affiliations:** 1 Nuffield Department of Population Health, University of Oxford, Big Data Institute, Old Road Campus, Roosevelt Drive, Headington, Oxford, OX3 7LF UK; 2 School of Epidemiology, Public Health and Preventive Medicine, University of Ottawa, Ottawa, Canada; 3 School of Epidemiology, Public Health and Preventive Medicine, University of Ottawa, Ottawa, Canada; 4 Population Health & Occupational Disease, National Heart and Lung Institute, Imperial College London, London, UK

**Keywords:** Measurement error, Continuous exposure, Nutrition, Statistical methods

## Abstract

**Background:**

Several statistical approaches have been proposed to assess and correct for exposure measurement error. We aimed to provide a critical overview of the most common approaches used in nutritional epidemiology.

**Methods:**

MEDLINE, EMBASE, BIOSIS and CINAHL were searched for reports published in English up to May 2016 in order to ascertain studies that described methods aimed to quantify and/or correct for measurement error for a continuous exposure in nutritional epidemiology using a calibration study.

**Results:**

We identified 126 studies, 43 of which described statistical methods and 83 that applied any of these methods to a real dataset. The statistical approaches in the eligible studies were grouped into: a) approaches to quantify the relationship between different dietary assessment instruments and “true intake”, which were mostly based on correlation analysis and the method of triads; b) approaches to adjust point and interval estimates of diet-disease associations for measurement error, mostly based on regression calibration analysis and its extensions. Two approaches (multiple imputation and moment reconstruction) were identified that can deal with differential measurement error.

**Conclusions:**

For regression calibration, the most common approach to correct for measurement error used in nutritional epidemiology, it is crucial to ensure that its assumptions and requirements are fully met. Analyses that investigate the impact of departures from the classical measurement error model on regression calibration estimates can be helpful to researchers in interpreting their findings. With regard to the possible use of alternative methods when regression calibration is not appropriate, the choice of method should depend on the measurement error model assumed, the availability of suitable calibration study data and the potential for bias due to violation of the classical measurement error model assumptions. On the basis of this review, we provide some practical advice for the use of methods to assess and adjust for measurement error in nutritional epidemiology.

**Electronic supplementary material:**

The online version of this article (10.1186/s12874-017-0421-6) contains supplementary material, which is available to authorized users.

## Background

Many exposures investigated in epidemiological research, such as physical activity, air pollution and dietary intake, are challenging to measure and therefore prone to measurement error. It has been suggested that efforts should be devoted to improving the measurement of an individual’s environmental exposure, the “exposome”, akin to the efforts that have been devoted to the “genome”, [1] and that international collaborations should be formed in order to translate the “exposome” from a concept to an approach that can be implemented in order to address public health issues [2]. An exemplar is diet and public health, about which there has been renewed controversy recently [3, 4]. The controversy is due in part to the difficulty of measuring nutritional and dietary exposures, and disentangling their effects because both specific exposures and their errors are inter-correlated.

### Measurement error in nutritional epidemiology

Two general types of measurement error are described in the nutritional epidemiological literature: random errors and systematic errors. Random errors are chance fluctuations or random variations in dietary intake that average out to the truth in the long run if many repeats are taken (i.e. the law of large numbers applies) [5]. Systematic errors are more serious as they do not average out to the true value even when a large number of repeats are taken. In epidemiological studies random or systematic errors can occur at two different levels: within a person or between persons. Thus at least four types of measurement error can exist and these are described in detail in Table 1.

**Table 1:** Types of measurement error

**Within-person random error:** This is the variation that is observed in exposure using a specific instrument when it is repeatedly measured in the same individual. A nutritional example would be the day-to-day variation in dietary intake reported using multiple 24-h recalls for an individual (assuming that it is possible to capture a single day’s dietary intake perfectly). The day-to-day variation may be random and thus results in an estimate of usual intake that is unbiased meaning that a person’s true usual intake is estimated accurately on average with several repeat measurements, although with some error.
**Between-person random error:** When error is random between individuals it results in an unbiased estimate of the mean usual exposure for the population of interest. Even with random measurement error within a person, it is possible to calculate an unbiased estimate for the population, by balancing out overestimation of some individuals with underestimation for others. With between-person random error the mean is estimated without bias, but the variance is inflated. In nutritional research this can be the result of using a single or a few repeat measurements of dietary intake per individual in the presence of within-person random error.
**Within-person systematic error:** Systematic errors are biases in the measurement of an exposure that consistently depart from the “true exposure” value in the same direction. Within-person systematic errors are systematic errors that are specific to an individual that are manifested as a positive or negative difference between an individual’s reported exposure. For example, some individuals may occasionally use dietary supplements which may lead to “systematic additive error,” indicating that a constant error is added to each person’s reported dietary intake. This could lead to over- or underestimation for all participants by the same amount. This directional difference (or intake-related bias) is usually constant within an individual and would remain regardless of how many repeat measurements are taken. Within-person systematic error may be related to individual characteristics, such as social/cultural desirability, that affects how a particular individual reports dietary intakes.
**Between-person systematic error:** Systematic errors in exposures can be additive or multiplicative. Additive between-person systematic error can occur when the dietary instrument of interest causes every measurement to be too large or too small by a constant amount from the truth. For example if the additive systematic error was negative each participants reported intake would be lower than their true intake using the dietary instrument of interest. Multiplicative between-person systematic error can occur when instead of reporting their true intake all participants report a fixed multiple of their true intake. This can be thought of as an intake-related bias where there is a systematic deviation from the truth due to a correlation between errors in the dietary instrument of interest and true intake. The attenuation (or flattened slope phenomenon) happens when both additive and multiplicative (intake-related bias) are present, which is typical in nutritional epidemiology. Person-specific bias is another type of between-person systematic error that may occur, if for example, a person that takes a dietary supplement every day – their average intake will be different from the predicted group-level flattened slope.

The situation where the only measurement errors are within-person random errors that are independent of true exposure with a mean of zero and constant variance is known as the “classical measurement error model” (Table 2) and, in the case of a single mis-measured exposure, its effect is always attenuation of the estimated effect size toward the null [6]. Thus, the magnitude of association is reduced but the statistical test used to estimate the dietary effect is still valid (i.e. the Type I error is unaffected), though its power is reduced. However, in the presence of covariates in the disease model which are also measured imprecisely, the effect of the main exposure could be biased in any direction due to residual confounding and, as a result, the statistical test becomes invalid [7, 8].

**Table 2:** Classical Measurement error model

The classical measurement error model assumes additive error that is unrelated to the targeted consumption, unrelated to other study subject characteristics, and independent of the corresponding measurement error in the dietary instrument of interest [103]. It is important that nutritional epidemiologists are aware of what sort of impact measurement error can have on diet-disease associations derived from even generally well conducted large-scale epidemiological studies. If there is a linear relationship between a single dietary exposure and the disease of interest, as in a logistic regression model, and this is also the case for a Cox regression or linear regression model, then the effect of classical measurement error is to attenuate the diet-disease association [37, 47]. This means that diet-disease associations such as log odds ratio, log hazard ratios or linear regression coefficients will be biased towards the null and a further consequence of classical measurement error in linear models is a loss of power to detect diet-disease associations. Classical measurement error in a multivariable exposure situation can bias the diet-disease associations in any direction, even in a linear regression model [47]. Other types of error that depend on the ‘true’ exposure (i.e. systematic error) or that depends on the outcome (i.e. differential error), may result in biases either away or towards the null in an unpredictable manner. [42, 47]	

### Dietary assessment methods

Many large-scale epidemiological studies rely on self-reported measures of dietary intake as this is the most cost-effective way to collect the information, but these are subject to error. Methods to assess dietary intake include i) food frequency questionnaires, that ask how often certain foods are eaten over a designated period of time; ii) 24-h recall are a memory based assessment method that asks about dietary intake over the past 24 h; iii) food diaries which prospectively record dietary data intake for a designated period; (iv) diet history and (v) checklist questions that assess one specific aspect of dietary intake.

The primary aim of many epidemiological studies is to obtain the “true dietary exposure” (which is usually defined as the habitual or long-term average dietary intake over a designated period of time), and establish whether this “true dietary exposure” is associated with a disease of interest. In order to facilitate this process the semi-quantitative food frequency questionnaire (FFQ) became popular [9]. An FFQ consists of a structured food list and a frequency response section on which participants indicate his or her usual frequency of intake of each food over a certain period of time in the past, usually the past year. The FFQ has a low participant burden, and thus it is possible to conduct repeated measures over time, which is important to capture long-term variation in diets. The FFQ usually has lower within-person variation than other dietary assessment methods described above because they are designed to assess long-term dietary intake, the exposure of etiological interest for most diseases [10].

### Gold standards and alloyed gold standards

A gold standard dietary assessment instrument measures the “true dietary exposure level” plus classical measurement error [7]. Multiple week diet records which require participants to record everything they eat or drink over the course of several weeks, are considered to be the “gold standard” for ascertaining self-report dietary information because unlike other methods described they do not rely on memory [10]. In some situations, biomarkers can be used as objective measures of dietary intake; these include recovery biomarkers which provide an estimate of absolute intake over a fixed period of time. Examples of recovery biomarkers are doubly labelled water for total energy intake and 24-h urinary nitrogen for protein intake, which provide a measure of intake based on a known direct quantitative relationship between intake and output [8].

Because multiple week diet records are seldom practical, and there are few available biomarkers, “alloyed gold standard” diet assessment instruments have been used to assess long-term dietary intake, such as multiple 24-h dietary recalls. Essentially, these are the best performing instruments under reasonable conditions, known to have some residual error but practical to use. Repeated 24-h dietary recalls involve a participant reporting all foods consumed in the previous 24-h or calendar day to a trained interviewer either in person or over the phone on multiple occasions over time. Although reliance on a participant’s memory leaves room for measurement error that is not necessarily classical, a well-trained interviewer can elicit highly detailed and potentially useful nutritional data. “Alloyed gold standard” biomarkers applied to the assessment of dietary intake includes i) predictive biomarkers and ii) concentration biomarkers. Predictive biomarkers are sensitive, stable, time-dependent, and show a dose-response relationship with dietary intakes. However, they may be affected by personal characteristics but their relationship with diet generally outweighs those factors. Examples of predictive biomarkers are urinary fructose, sucrose and dietary sugars. Concentration biomarkers are measured concentrations of specific compounds in blood (e.g. serum carotenoids, vitamin C and vitamin E) or other tissues (e.g. adipose tissue fatty acids). Unlike recovery biomarkers, concentration biomarkers do not have the same quantitative relationship with intake over a specific time period for all individuals in a given population due to between-subject variation in digestion, absorption, body distribution, synthesis, metabolism and excretion [8]. Thus, an alloyed gold standard dietary assessment instrument measures “dietary exposure level” plus non-classical measurement error.

### Reference instruments and calibration studies

As there is no universal method of assessing diet/nutritional exposures, and because of technological innovation, investigation of diet-disease relationships often have required, and increasingly require, the development of new instruments that can be used in large-scale epidemiological investigations. In principle, a measure of dietary exposure is valid if the dietary instrument measures what it purports to measure, which can be assessed if we can compare it to a reference instrument [11]. In the ideal scenario the new dietary instrument should be compared to a perfect reference instrument (that is a gold standard dietary instrument), that measures the “true dietary exposure”. In most situations, however, it is not possible to have an ideal or gold standard reference instrument and the new instrument for assessing dietary exposure is usually compared with an imperfect reference instrument (an alloyed gold standard), which is considered from previous research to be more accurate than the new dietary instrument. Thus, the relative validity (Table 3) of the new instrument compared with the reference instrument is investigated [11].

**Table 3:** Relative validity

In general terms, a study of ‘relative validity’ is one that compares the performance of two or more imperfect instruments, for example, food frequency questionnaire (FFQ) relative to other self-reported instruments, such as 24-h dietary recalls and food records [104]. The evaluation of a dietary instrument can therefore involve both the assessment of its measurement error structure and its correlation with the truth (i.e. its ‘relative validity’).
Often researchers will aim to assess the ‘relative validity’ of a new dietary instrument, such as a FFQ, by comparing its results with those obtained with a more accurate measure of food or nutrient intake. This can be in the context of the development of a new instrument, to test whether it provides improvement over currently used instruments, or for the use of an existing instrument in a different population from the one in which it has been developed. The development of any given FFQ is based on the dietary intake of a defined population during a specific period in time, and when these instruments are to be used in other populations, it is important to evaluate whether the instrument gives the same results when repeated on several occasions (the ‘reproducibility’) as well as its ‘relative validity’ in the new target population [11].

To quantify or make corrections for the effects of measurement error on estimated diet-disease associations, information is required additional to the main study data. Generally, random error can be addressed simply with replicate measurements of dietary intake using a specific dietary instrument for a sample of participants, while addressing systematic error requires an additional instrument, assumed to be free of systematic error, to be used as a reference. A smaller detailed study (we use the term “calibration study” to describe these) can be designed to obtain this additional information on random or systematic error in the dietary instrument of interest, in order for this information to be used to quantify or adjust for measurement error (see Table 4). The high participant burden and cost of keeping food records has limited their use in large-scale epidemiological studies. However, their ability to accurately ascertain detailed dietary information makes them useful as reference instruments for other dietary assessment methods in a calibration study. Because of their reliance on memory, FFQs may suffer from greater measurement error relative to 24- h dietary recalls and food records. Food records and 24-h recalls collected over several days can be used as reference instruments to reflect longer-term intakes (for certain nutrients, just a few days of diet records or 24-h recalls in a calibration study might be enough, provided the days are spread out over the entire reference period of the FFQ) [10]. The choice of reference instrument is therefore important and must be based on the judgement of the investigator and the research question. Choosing a particular dietary instrument as a reference means that the researcher is implicitly assuming that this dietary instrument is an unbiased estimate of the “true underlying dietary exposure” that they wish to measure.

**Table 4:** Types of calibration study

For the purpose of this report, we collectively refer to “calibration studies” to indicate studies that either (i) aim to assess systematic error by comparing a dietary assessment instrument with “true exposure” (or “gold standard” reference instrument) or with a known superior dietary instrument which may also be prone to its own measurement error as the reference instrument (an “alloyed gold standard”); (ii) aim to assess random error by taking repeat measurements using the same dietary instrument. Calibration studies can be “internal” if they are performed on a subsample of the main study, or “external” otherwise [46]. Calibration studies that use repeat measurements are common because under the classical measurement error model the error prone measurements of dietary intake are described as unbiased measures of ‘true’ exposure. This is due to the fact that under the classical measurement error model (i.e. errors in repeat measurements are uncorrelated) the average over a large number of repeated measurements would provide a good estimate of the ‘true’ exposure [7].	

Data collected from different types of calibration studies can provide information on the measurement error structure of the dietary instrument that can be incorporated in the analysis of the diet-disease association, to mitigate the impact of measurement error.

Different statistical approaches have been proposed to quantify and correct for measurement error, both in general and specifically for continuous dietary exposures. However, only one overview is available that has summarized all of the main issues and implications for policy decisions based on dietary associations but this review did not give a detailed description of the methods used [10]. We therefore undertook a systematic review to identify and appraise methods that have been applied in nutritional epidemiology to assess “true dietary intake”(defined as usual or habitual levels of a continuous dietary exposure), to assess different types of measurement error and adjust diet-disease associations for them.

## Methods

Studies were identified from the online databases Medline, Embase, BIOSIS and CINAHL, using the following search strategy up to the end of May 2016: [(*“diet” or “diet records” or “diet surveys” or “food” or “food preferences” or “food habits” or “food analysis” or “nutrition assessment” or “nutrition surveys” or “nutritional physiological phenomena”*) in subject headings or (*“food frequency questionnaire” or “FFQ” or “diet questionnaire” or “diet” or “24 h recall” or “24 h recall” or “24 h recall” or “weighed record” or “unweighed record” or “diet diary” or “food diary”*) in abstract or title] and (*“measurement error” or “mismeasurement”*) in abstract or title. We also searched the Dietary Assessment Calibration/Validation Register [12] and scrutinised the reference lists of relevant study reports and review articles, and by enquiring among collaborators and colleagues.

### Study identification and data extraction

Reports were potentially eligible for inclusion in this systematic review if they satisfied any of the following criteria: (1) they reported the development of a new method to investigate measurement error (2) they considered the issue of measurement error using some form of calibration study; (3) one or more methods were motivated by, and applied to, a real dataset. Using a pre-designed piloted data extraction form, data were extracted on: the statistical assumptions of the method [e.g. whether the method can deal with differential (error structure differs between groups of subjects in the study) or non-differential (error structure is the same between groups of subjects in the study) measurement error]; the reported increase in precision of the method compared with other possible methods of correcting for measurement error; the main and calibration study designs reported to be most appropriate for implementation of the method; whether the method can be implemented in standard statistical software. Based on our search strategy a single reviewer reviewed the titles, abstracts and keywords of every record retrieved. Full articles were retrieved for further assessment of eligibility for inclusion in the review by pairs of reviewers independently using the pre-designed extraction form and any disagreements were discussed with a third party. The full study protocol for this systematic review has been previously published [13]. This systematic review is reported according to the guidelines specified in the Preferred Reporting Items for Systematic Reviews and Meta-Analyses (PRISMA) statement [14].

## Results

**Fig. 1:**
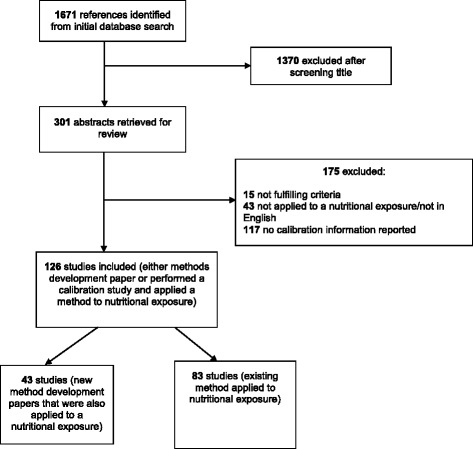
Screening and selection of included studies

Of 1671 potentially eligible articles identified in the search of the four databases, 126 studies met our eligibility criteria and had data extracted (Fig. 1). Of these, 43 were methodological (reported the development of a novel method and its application in a secondary analysis of a previously published nutritional epidemiological study) [Additional file 1: Table S1 gives the individual studies]; 83 were applied reports (i.e. they reported the application of an existing method) [Additional file 2: Table S2 gives the individual studies].

**Fig. 2:**
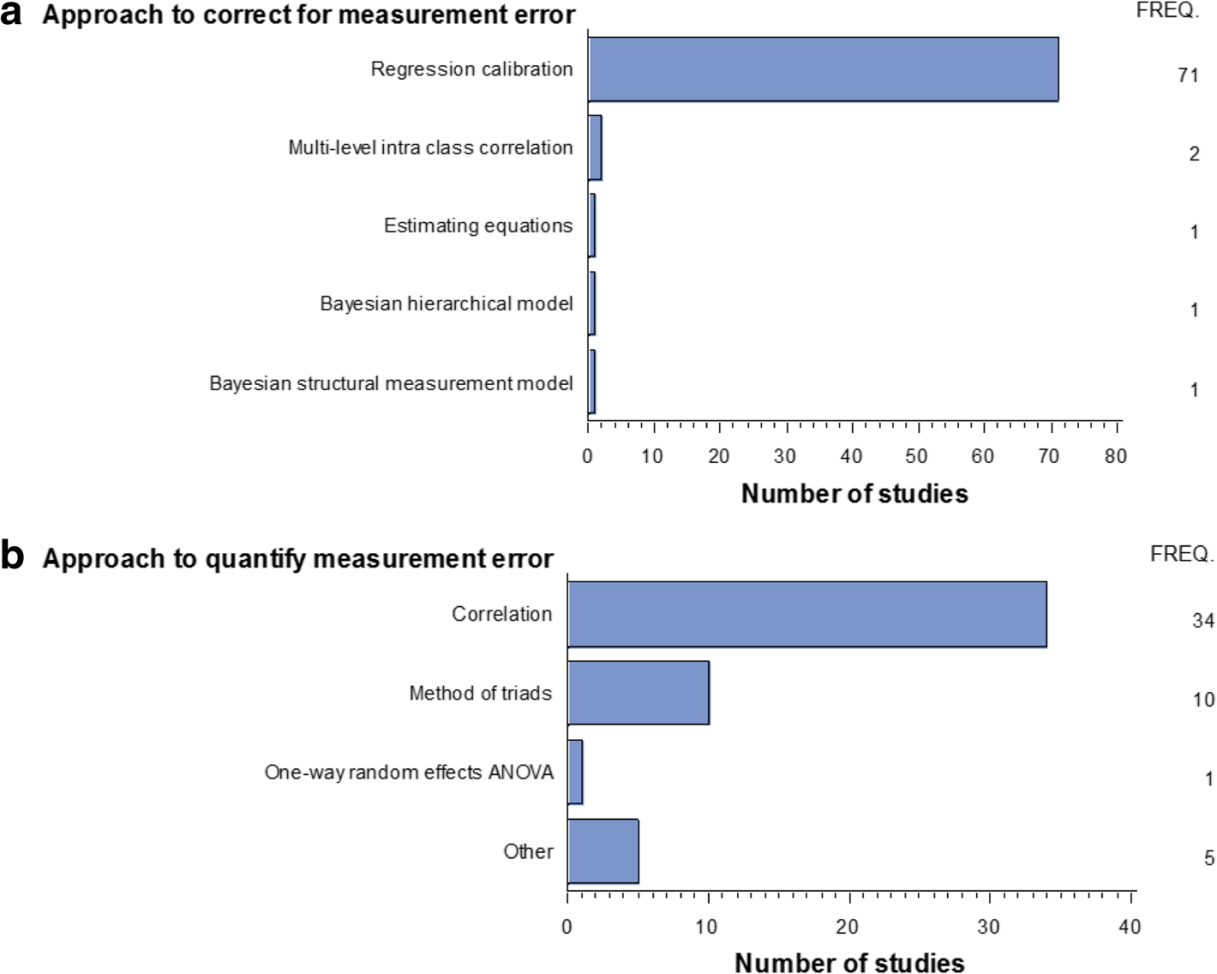
Summary of the main approaches used by studies included in the systematic review. **a** Approaches to correct for measurement error. **b** Approaches to quantify measurement error. FREQ.: Represents the number of studies that reported using the particular approach

We used a narrative approach for data synthesis and broadly classified the primary statistical approaches used in the 126 studies into two groups: a) approaches to quantify or assess the relationship between different dietary assessment instruments and “true intake”; b) approaches to adjust point and interval estimates of diet-disease associations for measurement error (Fig. 2).

### Approaches to quantify the relationship between different dietary assessment instruments and “true intake”

The dietary assessment instrument used most often in large-scale epidemiological studies is the Food Frequency Questionnaire (FFQ), which suffers from random measurement errors. The 50 studies included in this group aimed to assess the ‘relative validity’ of FFQs with alternative dietary assessment methods (e.g., 24-h recalls or diet records) by reporting correlations. The two main approaches identified were based on correlation analysis (34 studies) and method of triads (10 studies) [see Fig. 2b]. Table 5 provides a summary of the methods and their assumptions with reference to the original manuscripts and each of the methods is now described in more detail below.

**Table 5:** The method of triads and its extensions in the assessment of ‘relative validity’

Reference outlining the method	Classical measurement error model assumed	Requirements of calibration study	Relationship between reference instrument and dietary instrument of interest.	Aim of the approach
Method of Triads [105]	Yes	Three methods of assessment of dietary intake to be available (e.g. FFQ, 24-h dietary recalls and a biomarker)	The minimal statistical requirements are that the measurements from the three instruments are linearly related to the true intake levels and their random errors are statistically independent (i.e. uncorrelated).	To assess the ‘relative validity’ of dietary intake when the quantitative information was available for three methods (usually FFQ, 24-h dietary recalls and a biomarker).
Method of Triads Extension 1(MOTEX1) [2004] [27]	No	Superior or gold standard reference instrument available	The correlation between errors in the dietary instrument of interest and reference instrument can be non-zero (i.e. the errors are not statistically independent).	Aim to estimate the magnitude of correlations between errors in reference and the dietary instrument of interest (e.g. a FFQ).
Method of Triads Extension 2 (MOTEX2) [2005] [36]	No	Multiple dietary assessment methods required (e.g. self-reported instruments and biomarkers).	Three surrogate variables questionnaire (Q); M, and P where M and P are both instrumental (often biological) variables. No conventional reference instrument is required. M and P can be concentration biomarkers rather than recovery biomarkers.	To estimate the correlation between a dietary instrument of interest (Q) and true intake (T).
Method of Triads Extension 3 (MOTEX3) [2007] [35]	No	Multiple dietary assessment methods required (e.g. self-reported instruments and biomarkers).	No conventional reference instrument is required. Requires that error correlations between dietary estimates and biomarkers or between biomarkers be close to zero. M, and P are biomarkers with M being a direct measure of dietary intake and M and P are chose so that one has a long half-life and the other a short half-life.	Aimed to produce corrected estimates of the effects on an outcome variable of changing the true exposure variables by one standard deviation, a standardized regression calibration.

#### Correlation analysis

Correlation analyses were used by 34 studies included in this systematic review (Fig. 2b; Additional file 2: Table S2) to quantify the amount of measurement error. Correlation coefficients are commonly used to assess relative validity as they allow the presentation of the association between two continuous measurements as a single number. For example, in some situations a dietary questionnaire may be compared with a detailed diet history interview (or some other superior approach) administered at some later interval. Errors in the reference method should be uncorrelated with those of the main study dietary instrument being assessed; if these errors are uncorrelated then the correlation coefficients between the dietary intake as assessed by these two dietary instruments are usually underestimated [15]. In order to obtain a better estimate of the “true” correlation between the dietary intake as assessed by the two dietary instruments then a de-attenuated correlation can be computed using the approach summarized in Table 6.

**Table 6:** De-attenuated Correlation

When two measures are correlated, measurement error can lower the correlation coefficient below the level it would have reached if the measures had been free from measurement error. A de-attenuated correlation coefficient can be computed to correct for attenuation due to within-person variation if repeat measurements are available on the reference method. If for example, the dietary instrument was a FFQ and the reference instrument were multiple food diaries the de-attenuated correlation (ρ), under the assumption of a classical measurement error model, could be obtained by the formula: ρ = r √[1 + (wpv/bpv)*n]
Where r is the observed correlation; wpv is the within-person variance of the reference method; bpv is the between-person variance of the reference method; and n is the number of repeat measurements of the reference method [18]. Often variation due to daily energy intake is removed by adjusting for total energy using the residual method [106] prior to accounting for within-person variation in order to produce energy-adjusted de-attenuated correlations.

Two typical examples of analysis of correlations between two different instruments with subsequent calculation of de-attenuated correlation coefficients from studies included in this review are now briefly described. Horn-Ross et al. [16] measured four 24-h recalls in a 10 month study. The dietary recalls were spaced at three-month intervals beginning in early 2000. Two self-administered FFQs were completed. The first, covering usual dietary intake during 1999, was left with the participant following the first dietary recall and returned to the study office by mail. At the end of the study, a second FFQ, covering usual dietary intake during 2000, was mailed to the participant. The authors then calculated energy-adjusted de-attenuated Pearson correlations for the FFQ with 24-h recalls but did not mention performing any transformations prior to computing the correlations. Generally, the de-attenuated Pearson correlations ranged between 0.55 and 0.85 and the authors reported that these were consistent with other cohort studies [16]. Katsouyanni et al. [17] assessed the ‘relative validity’ of a 190-item semi-quantitative FFQ to be used in a large prospective study in Greece. Eighty participants completed two self-administered semi-quantitative FFQ spaced approximately 1 year apart, and within this 1-year interval they visited the study centre monthly and completed an interviewer-administered 24-h diet recall questionnaire. The authors reported that mean and standard deviations were calculated for all nutrient intakes from both FFQ and for the mean of the 24-h recall interviews. In order to account for non-normality all nutrient intakes were log-transformed prior to further analysis. The average correlation between the energy-adjusted nutrients measured by repeated 24-h recalls and the semi-quantitative FFQ was 0.46 for men and 0.39 for women. De-attenuated Pearson correlations varied for specific nutrients, ranging from 0.25 for beta-carotene and polyunsaturated fats to > 0.50 for saturated fats, cis-linoleic acid, calcium and phosphorus in both sexes combined.

As dietary variables are usually skewed toward higher values, transformations (such as logarithmic) to increase normality should always be considered before computing correlation coefficients. This has the advantage of reducing the influence of extreme values and of creating a correlation coefficient that is more interpretable. Alternatively, nonparametric correlation coefficients (e.g., Spearman) can be employed when one or both variables are not normally distributed [18]. Two studies in this review reported using Spearman’s correlation to assess the relative validity of dietary questionnaires in the Netherlands [19] and South Africa [20]. A similar study conducted in Germany performed transformations for non-normally distributed nutrients prior to analysis [21].

Willet has noted that a disadvantage of the correlation coefficient is that it is a function of the true between-person variation in the population being studied as well as of the accuracy of the dietary assessment method. Researchers who use correlations to assess relative validity of a FFQ with some other method should be aware that the correlation coefficient obtained is only generalizable to those populations with similar between-person variation as the test population, and the populations should be similar in terms of food items included, portion size and interpretation of the questions.

#### Method of triads

The method of triads (see Table 5), which has been proposed to assess the relative validity of three dietary assessment instruments and derive their validity coefficients, was used in ten studies included in this review [22–31]. The method of triads is often used with a FFQ (Q), a 24-h recall (I_1_) and one or more biological marker measurements (I_2_) [32]. The assumptions are that the measurements from the three instruments are linearly related to the true intake levels and that their random errors are statistically independent [11]. The assumption of independent random errors implies that correlations between any pair of measurements are entirely due to the fact that all measurements are related to the unknown ‘true intake’. Under these assumptions, the observed correlation between the three measurements Q, I_1_, and I_2_ can be written as products of the correlations of each of these three measurements with the “true” level of dietary intake of interest [33]. This method allows the comparison of food or nutrient consumption estimated by the three methods with the true (but unknown) intake by calculating a validity coefficient (the correlation between the dietary intake measured by the three methods and the true [unknown] dietary intake).

One of the problems with the method of triads is that implausible validity coefficients can occur. Validity coefficients typically lie between 0 and 1, but negative correlations and high random variation in the sample can lead to inestimable validity coefficients or validity coefficients greater than unity, a condition referred to as a Heywood case [25, 33]. The main reasons for a Heywood case are either violation of one or more assumptions of the method of triads, or random sampling variation [32]. Violation of the assumption of independence of random errors is more common, particularly when 24-h recalls or food records are used as the reference method since their errors are correlated with those of the FFQ.

Kaaks has suggested that using larger sample sizes and more accurate reference methods and biomarkers should reduce the chances of observing negative correlations [32]. More recent evidence from Geelen et al. [34] demonstrated that the method of triads can give misleading results when there are correlated errors between FFQ and 24-h recalls, because the method of triads is not able to correct for systematic errors (such as intake-related bias). Due to the correlation of errors between FFQs and 24-h recalls, some researchers have used the ‘validity coefficient’ for the FFQ as the upper limit, and the correlation coefficient of the FFQ with a biological marker as the lower limit, of the validity coefficient between FFQ and true intake [22, 23]. The ‘validity coefficients’ of a nutrient are not always comparable between studies as they may be estimated for different study populations or subgroups using population- or subgroup- specific reference methods [18].

#### Extensions to approaches based on method of triads

Three studies included in this systematic review were extensions to approaches based on the method of triads. Fraser and Shavlik [27] investigated how well data from a FFQ, a reference method, and a biological marker correlate with “true” dietary intake [referred to a MOTEX1 in Table 5]. They developed an error model that does not assume the classical measurement error model for either the reference method or the biomarker, and does not assume that the correlation between errors in the FFQ and reference method is zero. When they applied their proposed model to a calibration study, they found that correlations between reference method (24-h recalls) and true intake generally exceeded correlations between FFQ and true intake, which they suggested as supporting evidence that the reference method had better ‘relative validity’ than the FFQ. They also found that estimated correlations between errors in the 24-h recalls and FFQ were often much larger than zero, which violate the assumptions of the classical measurement error model. Generally for this approach to be applied a biomarker is needed in addition to dietary data (from two different dietary assessment methods) are required to allow calculation of correlations between estimated and ‘true’ dietary intakes [27]. In practice, however, sensitivity analyses are required in order to estimate the correlation due to the violation of these classical measurement error assumptions.

Two other methods that extend the originally proposed method of triads were proposed by Fraser et al. [35, 36] and incorporate two concentration biomarkers as well as a multiple 24-h recall or a FFQ [denoted by MOTEX2 and MOTEX3 in Table 5]. The authors analysed information from a calibration study that had used a FFQ, and collected biological data (biomarkers measured in blood or subcutaneous fat). They proposed using two biomarkers, of which the first was considered to provide an estimate of the unknown true intake of a specific nutrient, while the second was a biologic correlate only and did not directly measure the nutrient of interest [35, 36]. Thus, one of their examples used erythrocyte folate (considered to be a concentration measure) and vitamin E measured in the blood (a biologic correlate) in order to assess the relative validity of questionnaire measurements of folate intake [35]. The authors acknowledge that there are several limitations to their approach. First, there are few concentration biomarkers of nutritional intake, and even fewer recovery biomarkers. The available concentration biomarkers show a variety of half-lives and may require estimation in a variety of body fluids. Second, it is difficult to select an additional dietary biomarker that is a biological correlate of the intake of a given food or nutrient [35]. Third, it must be assumed that correlations between the errors of different dietary biomarkers are close to zero and the effect estimation must be in standard deviation units of the “true” variable. Fourth, the sample size of calibration studies needs to be large enough to ensure adequate precision of the validity coefficient using this approach - the authors recommend between 2000 and 3000 individuals representative of the main study population [35].

### Approaches to adjust estimates in diet-disease associations for measurement error

Two main approaches, one correlation-based and the other regression-based, have been used to adjust (“calibrate”) estimates in diet-disease associations assuming a classical measurement error model. The details of assumptions and implementation of these methods are summarized in Table 7 and are now described below.

**Table 7:** Intra-class correlation and regression calibration approaches to correct point and interval estimates assuming a classical measurement error model

Reference outlining the method	Requirements of the calibration study	Relationship between reference instrument and dietary instrument of interest.	Aim of the approach
Intra-class correlation [107]	Repeat measurements are available on the same individuals on the error prone dietary instrument.	No reference instrument is required just repeats of the dietary instrument of interest. However, the measurement errors in the repeated measures should be uncorrelated.	To be able to correct relative risk estimates and other regression slopes for bias. This approach can also be used to assess the reproducibility of a dietary instrument where a higher value indicates lower within-person variation.
Standard regression calibration [37, 42, 45]	External sample with gold standard reference instrument or repeat measures of the error prone dietary instrument of interest measure.	No correlation between the measurement errors in reference instrument and dietary instrument of interest.	To be able to correct relative risk estimates and other regression slopes for bias.
Multivariable regression calibration (MVRC) [42]	External sample with gold standard reference instrument or repeat measures of the error prone dietary instrument of interest measure.	No correlation between the measurement errors in reference instrument and dietary instrument of interest.	To be able to correct relative risk estimates and other regression slopes for bias.

#### Intra-class correlation

Two studies used the intra-class correlation as the main method of adjusting diet-disease associations for measurement error (Fig. 2a, Additional file 2: Table S2). In the situation where the measurement error is assumed to be strictly due to random within-person error, the intra-class correlation coefficient (ICC) can be computed based on replicate measures of the dietary exposure instrument, [37] and then the diet-disease association can be adjusted for it. The ICC represents the attenuation factor under the assumption of a classical measurement error model. When using repeat measurement data to compute ICCs to correct for measurement error, it is important to ensure that the calibration study is large enough to estimate ICC with reasonable precision [38, 39]. An example of the application of this method is the study of Horn-Ross et al. [40] that aimed to assess the association of alcohol intake with breast cancer. Participants were asked about alcohol intake in the year prior to the study start date, and at the end of the study one-year later asked about intake over the past year using an FFQ. The report found that ICCs for alcohol for pre- and post- FFQ ranged from 0.63 to 0.87. They reported that the corrected relative risk (per 20 g/1000 kcal/d) was 1.36 (1.03 to 1.51) compared to the uncorrected estimate of 1.25 (1.10 to 1.42) [16] for the association of alcohol intake with breast cancer. Another example is a study that aimed to assess the association of calcium intake with bone mineral content and bone mineral density. The study used repeat administrations of a youth specific FFQ in a multi-ethnic sample of children and adolescents and the ICC for calcium intake on a log-scale was found to be 0.61 [41].

#### Regression calibration

In this systematic review 71 studies used a linear regression calibration approach (referred to as standard regression calibration) in order to correct for measurement error or some variant of classical regression calibration (Fig. 2a; Additional file 1: Table S1; Additional file 2: Table S2; Table 7). Rosner’s linear regression calibration involves a regression of the reference measurement against main study measurement [37, 42]. The fitted values from the regression of the reference method against the main study dietary method should be a good estimate of the expected “true dietary” intake. Using these fitted values in place of the measured dietary intake in the main study should produce the “regression dilution” corrected estimate. This linear approximation approach is equivalent to the estimation of the regression coefficient of disease outcome on true dietary intake. The regression dilution correction factor b_RC_ is obtained by dividing the ‘naïve’ regression coefficient obtained by regressing the disease-outcome on the measured exposure in the main study by the regression coefficient obtained from a regression of true exposure measurements (reference method) on crude exposure measurements (based on the main dietary instrument) in the calibration study [7]. This is the classical linear regression calibration approach but it has also been argued that it may be more appropriate to regress the crude measurement on the more precise (i.e. the reference measurement) [43].

Linear regression calibration is by far the most common approach to correct for measurement error in dietary studies (Fig. 2a). In addition to the assumption of the classical measurement error model, linear regression calibration also assumes a linear relationship between disease outcome and true exposure, between reference method and main study method, and between disease outcome and main study method. These assumptions are only valid when the distributions of true exposure and measurement errors are normal. The method is tolerant to a modest degree of non-normality, but it would not be expected to perform well with grossly non-normal distributions such as those that may arise, for example, for intakes of some micronutrients (such as dietary carotenoids and tocopherols) [11]. Often, to get a better approximation for linearity, normality, or non-constant error variance, the dietary data are transformed, e.g. by taking logarithms. For example, the study of Prentice et al. [44] used urinary recovery biomarkers to correct FFQ assessments for measurement error, and examined absolute energy and protein consumption in relation to cardiovascular disease. Urinary recovery biomarkers of energy and protein were obtained from a subsample of 544 women, with concurrent FFQ information. The authors used simple linear regressions with log-transformed values for both the biomarker and the FFQ assessments in order to obtain an estimate of the correction factor b_RC_. The authors applied similar approaches to a more extensive series of dietary associations with chronic disease endpoints in a subsequent report [44].

Linear regression calibration also assumes that errors are non-differential with respect to outcome, which is a reasonable assumption in most prospective cohort studies but less secure in case-control studies that are not nested within a cohort. As mentioned earlier it also assumes uncorrelated errors between the dietary assessment method under investigation and the reference method.

For a binary outcome, where logistic regression analyses may be used the conditions for regression calibration are satisfied if the disease is relatively rare (< 10%), the odds ratio small (the odds ratio is a good estimate of the relative risk for rare diseases), and the measurement error small [2]. Rosner and colleagues have extended the regression calibration method to multiple logistic regression that accounts for both random and systematic within-person measurement error. Furthermore these methods provides tests and confidence limits for the association of interest that incorporate both the uncertainty in the estimate of the odds ratio (relative risk) from the main study and the uncertainty in the estimation of the correction factor [37]. The latter component is important because when calibration studies are small the degree of correction applied to the observed relative risk itself has error. As this method is based on a multiple logistic regression model, measurement error can be accounted for while simultaneously adjusting for confounding by other variables [18].

As an extension of the standard linear regression calibration method, again in the framework of the classical measurement error model, to deal with multiple covariates measured with error, Rosner and colleagues developed multivariable regression calibration (MVRC, Table 7), assuming either strictly random within-person error [45] or combinations of random and systematic error [42]. The latter approach is a multivariable extension of the linear approximation approach and requires a calibration study with all of the important covariates measured simultaneously. The method aims to take into account not only the error in the measured variables but also the correlation between the errors. This approach has also been applied to multiple linear regression and Cox regression models [46].

Rosner’s linear regression calibration approach is an indirect method of correction for measurement error. In an alternative direct approach to regression calibration first described by Carroll [47], the reported dietary intakes used as explanatory variables in the risk model are directly replaced by the expected values of the true usual intake predicted from the reported intakes and other important factors (such as confounders) that are included in the risk model. This expected value of true usual intake is usually obtained from a calibration study and produces an approximately unbiased estimate of the true relative risk for a dietary intake under the assumption of a classical measurement error model. In Carroll’s approach there does not have to be a linear relationship between the expected value of the true usual intake and the outcome of interest [47]. This is an important extension to Rosner’s method as sometimes linear regression calibration may not be able to obtain the optimal prediction of usual intake and a nonlinear model may be more appropriate [6].

#### Extensions to linear regression calibration to address departures from the main assumptions

Using “correct” reference instruments (“gold standards”) in regression calibration provides an unbiased estimate of the correction factor b_RC_, but using imperfect reference instruments will produce biased estimates of b_RC_. Several reports in this systematic review have conducted sensitivity analyses to assess and quantify the impact of the use of imperfect reference instruments and/or departures from the classical measurement error model on regression calibration. It should be recognised that some of the assumptions made in these approaches, as described below, are difficult or impossible to test directly, so investigators have suggested using sensitivity analyses or simulation-based analyses to approach these issues. Table 8 provides a summary of the methods that aim to address departures from the main assumptions of regression calibration and the key points are now described.

**Table 8:** Regression calibration based methods that do not assume a classical measurement error model

Reference outlining the method	Requirements of the calibration study	Relationship between reference instrument and dietary instrument of interest.	Aim of the approach
Person-specific bias adjusted regression calibration (PSBRC) [52]	Superior or gold standard reference instrument available.	An estimate of the person-specific bias in the reference measure and its correlation with systematic error in the FFQ is required.	To be used as sensitivity analysis in order to assess the impact of varying pre-specified ratios of the variance of the person-specific biases in a reference instrument and FFQ and the correlation between these biases.
Flawed reference instrument adjusted regression calibration (FRIRC) [50]	Internal or external sample with superior or gold standard reference instrument available.	Extension of PSBRC where the model assumes for both the FFQ and the dietary report reference instrument, group-specific biases related to true intake and correlated person-specific biases can be estimated.	To be used as a sensitivity analysis in order to assess the impact of additional complexity of both group and person-specific biases.
Biomarker and alloyed gold standard regression calibration (BAGSRC) [51]	Internal or external sample with superior or gold standard reference instrument available	Model assumes that there is a correlation between the “alloyed gold standard” and the level of exposure using dietary instrument of interest. If a third method of exposure assessment (biomarker) is available and it is reasonable to assume that the errors in this method are uncorrelated with the errors in the other two exposure assessment methods.	Estimate the bias in the standard regression calibration due to the correlation between alloyed gold standard and the level of exposure using dietary instrument of interest. Derive estimates of the correlation between the errors in alloyed gold standard and exposure assessment using biomarker data.
Auxiliary Information regression calibration (AIRC) [53, 54]	Internal or external sample with superior or gold standard reference instrument available (if a biomarker –then replicates)	The models account for correlated errors in the FFQ and the 24-h diet recall and random within-person variation in the biomarkers.	To be used as a sensitivity analysis in order to assess the impact of correlated subject-specific error on correction factor.
Episodically consumed foods regression calibration (ECFRC) [59]	External sample with superior or gold standard reference instrument available	Model assumed that a food is reported on the 24HR as consumed on a certain day if and only if it *was* consumed on that day. Also that the 24HR is unbiased for true usual intake on consumption days.	To predict an individual’s usual intake of an episodically consumed food and relating it to a health outcome.
Never and episodic consumers (NEC) model [88]	A subset of the population has repeat measurements of dietary instrument of interest.	Assumes that food record measurements are subject only to random within-person variability. The observed food record measurements are unbiased estimates of “true intake”. Nonzero food records measurements to be normally distributed on a transformed scale.	To predict an individual’s usual intake of an episodically consumed food whilst incorporating never consumers and relating it to a health outcome.

#### Departures from assumptions of classical measurement error in the reference instrument

There is abundant evidence that food record measurements are biased estimates of ‘true intake’, and that the error in food record measurements depends on both the level of ‘true’ exposure and person-specific errors. [48, 49] Kipnis et al. [50] define these sources of error as “group-specific bias”, common to all persons with the same “true dietary intake”, and person-specific bias, the group-specific bias together with the effects of personality, social and cultural influences on the reporting of dietary intake. Both are specific forms of systematic error and result in violation of the assumptions of the classical measurement error model. They also point out that these biases are part of within-person systematic error and will be reproduced in repeated measurements on the same individual.

Estimates of group-specific biases are affected by the choice of reference instrument. If the reference instrument is flawed (e.g. an alloyed gold standard with measurement errors related to true intake and not independent from the errors in the instrument under investigation), then this can bias the estimate of the correction factor. Kipnis et al. [50] investigated the impact of using a flawed reference instrument in standard regression calibration (FRIRC). Their results suggested that group- and person-specific biases existed in both the FFQ (instrument under investigation) and the weighed food record (flawed or imperfect reference instrument), and that the person-specific biases of the two measurements were correlated. They also suggested that the use of an imperfect reference instrument could lead to underestimation of the true effect by about 50%, with an approximately 2-fold inflation of the sample size required to detect the ‘true’ effect.

#### Use of biomarkers in addition to dietary instruments in regression calibration

Both recovery and concentration biomarkers have been used as reference instruments in four studies included in this report [51–54], and the implications of their inclusion have been explored via sensitivity analyses. An approach proposed by Speigelman et al. [51], described here as biomarker and alloyed gold standard regression calibration (BAGSRC), uses an imperfect reference instrument, a cruder dietary instrument and a biomarker. An additional assumption is that the errors in the biomarker are uncorrelated with the errors in both imperfect reference instrument and cruder instrument. These authors presented an example that assesses the measurement of dietary intakes of vitamins A and E. The semi-quantitative FFQ was the cruder instrument, the imperfect reference instrument was based on two diet records, and a biomarker for each of vitamins A and E intake were plasma concentrations of total carotene and α-tocopherol respectively. The findings of their study suggest that this approach is robust when the errors in the biomarker are uncorrelated with the self-reported measures, and can be used as an extension to standard regression calibration when a suitable biomarker is available [51].

Kipnis et al. [52] considered a measurement error model which takes into account both correlated person-specific biases and with-person measurement errors. This model incorporates the models of Freedman et al. [55] and Speigelman et al. [51] as special cases. However, when using a calibration study with an imperfect reference instrument the correction factor cannot be estimated as there are too many parameters in the statistical model, and therefore Kipnis et al. [52] had to assign fixed values to some parameters in order to make their models statistically identifiable. In the hypothetical scenarios considered, these authors found that the estimated relative risk using standard regression calibration could be dramatically underestimated when person-specific biases are highly correlated.

A more general version of BAGSRC was also described by Spiegelman et al. [53] and Preis et al. [54], here referred to as Auxiliary Information Regression Calibration (AIRC) (Table 7). The paper by Speigelman et al. [53] derived an explicit expression for the bias in the correction factor, and describes the magnitude and direction of bias in the relative risk estimate obtained through standard regression calibration as a function of the correlations of subject-specific biases and within-subject errors in the two measures being compared in the calibration study. To make the application of the Kipnis et al. [52] model feasible, they proposed two minimally sufficient external calibration study designs, each of which fully identifies the correction factor and other parameters of interest. They proposed a “replicated biomarker design” that includes information on dietary instrument of interest (Z), alloyed gold standard measure of exposure (G) and biomarker (W). They also proposed an augmented design that uses both a biomarker and an instrumental variable (a variable that is correlated with the true exposure X, and uncorrelated with the random and systematic error components in G and Z). They considered the impact of missing data on these new study designs. This was because more than two methods of exposure measurement are needed, some of which must be replicated, and there may be variable numbers of replicates within participants, and different patterns of missingness of the multiple modes of measurement among the study participants. Pries et al. [54] sought to estimate the correction factor for total energy intake, protein and protein density in the calibration and main studies. They used repeat-biomarker measurement error models, which account for both correlated errors in the FFQ and 24-h recalls and random within-person variation in the biomarkers. They further assumed that the diet records or the 24-h recalls were unbiased, while the biomarker was assumed to be biased in order to accommodate the fact that most biomarkers available to nutritional epidemiologists are of concentration rather than of recovery [54]. The authors concluded that under certain measurement error models failure to adequately account for within-person variability in the assessment method that is assumed to be unbiased (i.e. the reference method) can falsely lead to an appearance of correlated errors and to underestimation of the FFQ’s ‘relative validity’ [54]. However, it was later argued that the authors had erroneously concluded that within-person variation was underestimated as their model had assumed that the recovery biomarkers used (doubly labelled water and urinary nitrogen) were biased which in fact is more likely to be the case for concentration biomarkers, where this model had been previously applied [56]. Keogh, White and Rodwell [57] used a concentration biomarker to assess the levels of fruit and vegetable intake, and their findings were that the estimated effects of error in self-reported measurements was highly sensitive to model assumptions. They concluded that making the incorrect assumption of a classical measurement error could result in a large overcorrection for the effects of measurement error [57].

#### Incorporating episodically consumed foods in regression calibration

Four reports included in this systematic review attempted to assess the impact of episodically consumed foods on linear regression calibration estimates [58–61]. Willet has suggested that systematic between-person errors in dietary intake are likely to be frequent and can have many sources [18]. One source is episodically consumed foods, defined as foods not consumed every day by most people. Dietary assessment of episodically consumed foods gives rise to non-negative data that have excess zeroes (due to infrequent consumption) and measurement error [58]. Often, within-person random error in the 24-h recalls intake of episodically consumed foods is dependent on the individual mean and has a skewed distribution, violating the classical measurement error model assumptions. The most common technique has been to transform the data, so that the dietary intake measures more closely follow the classical measurement model with normally distributed errors. Kipnis et al. [59] incorporated episodically consumed dietary components (see ECFRC, Table 8) and assessed the impact using a nonlinear regression calibration approach. In their model, the probability of consumption for an individual may be arbitrarily small but always positive, thus allowing for any finite number of days with zero intakes, but not incorporating never-consumers (if they existed). Their model was in two parts: in the first part, consumption probability is modelled using mixed effects logistic regression; in the second part, the measurement error for the non-zero reported intake is modelled using a *nonlinear* mixed effects approach [59]. In addition, Kipnis et al. [59] proposed an extension to the method described by Tooze et al. [58] to predict individual usual intake of such foods and to evaluate the relationships of usual intakes with disease. One feature of their proposed method is that additional covariates that are potentially related to usual intake may be incorporated in order to improve the estimates of usual intake and of diet-disease associations. Due to measurement error, the naïve approach (using simple individual means of several 24-h recalls) grossly underestimates the true value, as expected theoretically. In their simulations, however, although the bias of their proposed method was smaller, the precision was poorer (i.e. wider confidence intervals which is a feature of correcting for attenuation) than using the naïve approach [59]. Agogo et al. [60] used a simulation study to assess a two-part model with part 1 assuming a logistic distribution and part II assuming a gamma distribution and required a single replicate of the dietary assessment instrument (a 24-h recall). This model was closely related to those already described by Tooze [62] and Kipnis [59] and the authors report that transforming the dietary assessment data to an appropriate scale was found to have the largest influence on model performance [60].

Keogh and White [61] describe a three part measurement error model that extends the two-part models described by Kipnis [59] and Tooze [58] above, with the third-part accounting for never consumers. They call their model the “never and episodic consumers (NEC, Table 8) model” that allows for both real zeros (never consumers), and excess zeros (episodic consumers of some foods), and requires repeat measurements. In the most realistic situation, where repeat measurements are available on only a subset of participants, the authors found via simulation that the NEC model appeared to be superior to classical regression calibration in terms of reduced bias [61].

#### Other approaches

In this systematic review there were several other approaches that were identified in the literature to deal with measurement error and these are summarized in Table 9 and an overview of these methods is now briefly given.

**Table 9:** Other approaches in this systematic review used to assess the impact of measurement error

Reference outlining the method	Classical measurement error model assumed	Requirements of calibration study	Relationship between reference instrument and dietary instrument of interest measure.	Aim of the approach
Simulation Extrapolation (SIMEX) [63]	Yes	External sample with two concentration biomarkers and internal sample with repeat measurements of the FFQ were also used.	Assumes random within-person error for FFQ and that concentration markers are uncorrelated.	To assess the impact of measurement error in nutrient intake as assessed by a FFQ when concentration biomarkers are also available.
Structural equation modelling [64]	Approach can be used with and without assuming a classical measurement error model.	Superior or gold standard reference instrument available with repeat measurements.	Varied the assumptions of the relationship of the reference instrument with the dietary measure.	Aimed to assess the different types of error (either random or systematic), and within or between individuals-that may occur in dietary intake measurements. In addition to demonstrate that the inclusion of biomarker data can allow the estimation of the average magnitude of these errors even if random errors of repeat measures of the reference instrument are correlated.
Moment Reconstruction (MR) [65]	No	Internal sample with gold standard reference instrument available.	Assumes that disease D, true exposure (X), exposure based on dietary instrument of interest (Z) and biomarker (M) are multivariate normal distributed.	As a sensitivity analysis to show that other “substitution methods” have advantages over standard regression calibration when the measurement error is differential (i.e. *error* is related to disease outcome D).
Imputation (IM) [65]	No	Internal sample with gold standard reference instrument available.	Assumes that disease D, true exposure (X), exposure dietary instrument of interest (Z) and biomarker (M) are multivariate normal distributed.	As a sensitivity analysis to show that other “substitution methods” have advantages over standard regression calibration when the measurement error is differential (i.e. error is related to disease outcome D).

One report [63] used Simulation Extrapolation (SIMEX), which is similar to regression calibration but uses a simulation-based approach rather than a classical regression approach. The algorithm performs multiple iterations whilst adding a small known amount of error at each successive iteration, and re-estimates the parameters each time. A trend in the effect of the measurement error is then estimated, and extrapolation made to the case where there is no measurement error. However, SIMEX can only be applied if the measurement error is classical and additive or multiplicative (Table 9). Two reports demonstrate that the four main types of measurement error (see Table 1) can also be incorporated in Structural Equation Modelling (SEM) [63, 64]. SEM considers the measurement error process as a latent variable problem, with the unknown “true exposure” estimated on the basis of a calibration study. The SEM approach is a method for estimating the measurement error structure using additional instruments, with at least one dietary instrument assumed to have no systematic error. However, the approaches of Speigelman et al. [53] and Preis et al. [54] require only that the model is correctly specified, whereas the SEM approach is more restrictive as it requires full multivariate normality of all random variables in the model.

All of the approaches considered in the review so far have assumed that the measurement error in non-differential (i.e. the error is the same for those with and without the outcome). Two other approaches, moment reconstruction and imputation, may have an advantage over standard regression calibration when there is differential measurement error (error whose magnitude or direction is different for individuals who have the outcome), such as in a case-control study where cases and controls may recall their dietary intake differently [65]. First, the moment reconstruction (MR) method was proposed by Freedman et al. [66] as a method for correcting error in univariate continuous exposures, and requires an internal calibration study where the ‘true’ exposure is measured for some individuals where their disease (or outcome status) is known. The estimates of the true exposure derived from the MR method can be used directly in the diet-disease model, and it has been found that this results in unbiased estimates of linear exposure-disease associations [65]. Second, as measurement error can also be conceptualised as a missing data problem in that the “true exposures” are missing, imputation methods have sometimes been used. Multiple imputation (MI) was first introduced by Rubin and has become a widely used approach for dealing with missing data for a variety of epidemiological study designs [67]. Freedman et al. [65] suggested using MI to correct for measurement error in continuous exposures, by treating the true continuous exposure data as missing. The key idea is that values of the true exposure are imputed by drawing a random value from the distribution of the true exposure conditional on all observed data, including the outcome (or disease status). Multiple imputation methods involve creating a number of imputed datasets that would then be available for standard statistical analyses that correct for measurement error, before combining the estimates from each analysis into a final result, using an approach first developed by Rubin [67]. If a calibration study within which the true exposure was available, (via a “gold standard reference instrument”), estimation of the true exposure distribution would follow a similar process to a standard missing data analysis procedure. If the calibration study was based on repeat measurements, then the procedure requires the estimation of the true exposure conditionally on repeat measurements of the error-prone exposure, and the covariates measured without error. The authors demonstrate that if MR and MI are used when the measurement error is in fact non-differential, then these methods will in general result in a loss in efficiency relative to standard regression calibration [65].

#### Impact of departures from classical measurement error model on statistical power

All of the approaches identified in the systematic review were generally developed and employed to assess the impact when the assumptions of standard regression calibration are not satisfied. In addition to the bias that may ensue in the estimation of diet-disease association, Fraser and Stram [68] demonstrate via simulation that correlated errors can lead to an added loss of statistical power (reduced to as low as 11% in their simulations) if small (< 100 participants) calibration studies are used to adjust point and interval estimates using standard regression calibration and there are few events (for example ≤250) in the main cohort. Fraser and Stram [68] conclude that if there are modest correlations (≤ 0.5) between the reference instrument and the cruder instrument, useful gains in power accrue (up to 5-fold) with calibration study size up to 1000 participants with a reasonable number of events (for example ≥2500) in the main study. More recently, Fraser and Stram [69] considered the impact of non-Gaussian distributed dietary intake data on standard regression calibration, and they reported that poor fit in the calibration model: a) does not produce biased calibrated estimates when the “disease” model is linear; b) it produces little bias in a nonlinear “disease” model if the model is approximately linear; and c) will adversely affect statistical power, but this could be alleviated by attempting some of the more complex sensitivity analysis models for dealing with departures from the classical measurement error model outlined in this report [69].

## Discussion

Most of the literature addressing the issue of measurement error in nutritional epidemiology is based on the assumption of random within-person error. This is due to the fact that a) systematic error is much more difficult to measure and b) most of the statistical theory and methodological developments have been based around the assumption of random error. In this systematic review, we found that the most commonly reported statistical methods in nutritional epidemiological studies to quantify measurement error were correlation-based methods. With regard to assessing and correcting for measurement error, by far the most commonly used method was linear regression calibration. A key issue is that the assumptions of the method are satisfied.

Based on the evidence identified in this systematic review, we now propose useful points to consider when the intention is to use a calibration study to either assess a new dietary instrument, quantify measurement error or implement measurement error corrections of diet-disease associations.

### General design of a calibration study

A key design issue, which has been addressed by most papers reviewed, is that the calibration study has to be representative of the main study, and this is most likely to be achieved using an internal calibration study based on a random sample of the main study. Depending on the type and purpose of the calibration study, Caroll et al. has suggested that one may use large sample sizes and few food records per individual or smaller samples and more records per subject [70].

### Calibration studies to assess and correct for measurement error

In the vast majority of the studies reviewed, a dietary questionnaire measurement was compared to a more detailed and reliable reference instrument, such as multiple 24-h recalls or food diaries [71]. The utility of comparing a crude dietary instrument with a reference instrument depends on the important statistical assumption that the reference instrument follows the classical measurement error structure, i.e. within-person errors for the reference instrument are strictly random. When errors in the crude and reference instrument are dependent, standard regression calibration will under-correct the diet-disease association. Thompson et al. [72] report that, due to correlation of errors in FFQs and self-reported reference instruments such as the 24-h recalls, the correlations and correction factors observed in most calibration studies tend to overestimate FFQ performance. Other researchers have quantified the direction and magnitude of the bias, and have concluded that bias appears to lead to small overestimation in realistic examples [48, 54].

It has been recommended that, when possible, biomarkers should be incorporated into the calibration study as objective measures of intake [73], under the assumption that the measurement errors in biomarkers are independent of the measurement errors from self-reported dietary measurements, [74] although this has never been verified in practice. Keogh et al. [57] used repeated biomarkers to quantify the bias due to measurement error for a self-reported dietary intake from FFQs and food records. They caution that the magnitude of the correction factor is highly sensitive to the model assumptions and that sensitivity analyses that assess the impact of varying these assumptions are essential [57]. In theory additional types of biomarkers, for example metabolomic profiling, may also be incorporated into the calibration study [73]. Unfortunately, only a limited number of recovery biomarkers have so far been identified as able to reflect an individual’s intake with a high degree of accuracy.

Careful consideration of the assessment period and the use of updated dietary information are necessary, particularly if the interest is long-term usual dietary intake since diet-disease relationships may change over time. It is therefore important that the comparison method employed is able to reflect the longer time frame. If there is likely to be medium term variation due to seasonality, then it would be sensible to have multiple measurements throughout the year. Biomarkers can also change over time, so it is important to obtain repeat assessment of these too if possible. It has been demonstrated that if a FFQ is being used with a diet record as the reference instrument and a suitable biomarker exists, and the error in the biomarker is independent of the error in the FFQ and the diet record, then the methods of triads can produce an estimate of the correction factor that is unbiased provided that there are replicate biomarker data on at least a subsample of calibration study subjects [31].

### Calibration studies to assess the ‘relative validity’ of a new dietary instrument

Calibration studies can also be used in the development of a new measurement instrument to test whether it provides improvement over currently used methods, and several studies in this report were of this type. In this context, the ‘relative validity’ between the current and the new measurement instrument would be of primary interest [75]. However, the use of correlations may not be the optimal approach in order to assess ‘relative validity. An improvement is to assess the level of agreement (between say a FFQ and 24-h dietary recalls), as proposed by Bland and Altman [76]. In this approach, the difference between mean intakes estimated by the FFQ and 24-h dietary recalls is plotted against the average of mean intakes by the two methods for each participant and nutritional measure. The Bland-Altman approach of plotting difference against average only works if the average is an unbiased estimate of the truth. In our example it may be better to plot the difference of FFQ and 24-h recalls against the average of several 24-h recalls (the reference instrument and our best estimate of the truth). If the data follow a normal (Gaussian) distribution it would be expected that the mean difference (bias) lies between ±2 standard deviations, and a lack of heteroscedasticity (or non-constant variability) indicates that the magnitude of error does not vary with the range of measurement [76]. These analyses would need to be performed for all nutritional measures (such as macro- and micro-nutrients). Willett has suggested that the Bland-Altman method may be too cumbersome as it requires considerable knowledge about the typical absolute values and between-person variation, which differ from nutrient to nutrient [18]. He suggests an alternative, but related method, that involves the calculation of the standard deviation of the “residual” from the regression of the true measure (estimated using the reference method) against the dietary assessment instrument. This “residual” would then represent the variation in true intake that is not accounted for by the dietary assessment instrument [18].

For an assessment of ‘relative validity’, there is always the decision as to whether or not the new dietary instrument is considered to be a satisfactory. Nelson and Margetts note that there are no hard or fast rules as to what constitutes ‘satisfactory’ correlation, as it is usually dependent on the sample size of the study [11]. As Willett has suggested, and we concur, because no single method for relating dietary intake to a measure of true intake conveys all the available information, it is probably best to present the data in several ways. At a minimum, the means and standard deviations of the reference method and dietary instrument of interest plus their correlations should be provided [18]. These may be supplemented with other data such as Bland-Altman plots or the residual regression approach.

### Number of replicate measures and sample size of the calibration study under the assumption of random within-person measurement error

Many of the methods considered in this report assume that the errors in dietary intake are due to random within-person variation, If the aim of the calibration study is to correct for measurement error rather than to assess relative validity then to minimize the correlation between errors on the different occasions, the repeated measurements should be well separated in time. In addition, the measurement of dietary intake via more than one instrument (as different instruments have different strengths and weakness) [77] in a subsample, is essential [7]. It has been recommended that fewer repeats on more individuals provide greater statistical efficiency [78, 79]. Some have argued that in most settings, cost-efficient designs of FFQ calibration studies do not require more than four or five 1-day diet records per participant in order to estimate the true long-term dietary intake, particularly when the costs of collecting any subsequent measure is the same as for the initial dietary measure [79]. Rosner and Willett [80] also considered the cost of collecting replicate information, and found that generally there was no justification for more than two to five measurements per participant, assuming that the total cost of the study was proportional to the total number of observations. Taking the average of multiple reference instrument measurements per participant (e.g., multiple food records or multiple 24-h recalls) can increase the correlation (i.e. the relative validity’) between the reference instrument and the questionnaire [74]. However, multiple 24-h recalls per participant can lead to high burden on the participant and poor quality information. Moreover, averages over a few days are not an adequate representation of a participant’s usual intake [81]. Thus computing the de-attenuated correlation will also increase the correlation coefficient under the assumption of a classical measurement error model. Wong et al. [82] provide examples of sample size calculations for the design of such calibration studies based on the within-person correlation of the reference instrument. Park and Stram [83] noted that the percentage of individuals from the main study that are required to participate in the calibration sub-study decreases in size as the correlation between true and observed exposure increases, that is as measurement error becomes smaller. However, others have suggested that optimal balance of repeats and individuals depends on the primary aim of the calibration sub-study (either to compare a new with an old instrument for ‘relative validity’, or specifically to correct for measurement error in the main study), as well as the within-person variation of the repeated measurements [75]. The effect of measurement error is also reduced when the variance of the true exposure is large relative to the variance of the error [84, 85], since this brings the correction factor closer to 1 and therefore reduces bias on average. The easiest way to do this is to ensure sampling of a wide range of exposures. Even though several studies in this systematic review wanted to use the calibration study information to correct for measurement error, none of these studies reported details of any formal sample size calculations for the size of the calibration study. Kaaks and colleagues have presented some more formal approaches based on the precision required, the cost of conducting the calibration study [78], and Carroll et al. have also made some sample size recommendations that take into account the instrument used (e.g. 24-h recalls or multiple-day food records) [70]. Willet has suggested that because the most important use of a calibration study is to correct observed diet-disease associations for measurement error, an approach for choosing an appropriate sample size would be to consider the implications of various sample sizes on the corrected effect size estimates and their corrected confidence intervals [18]. In particular calibration studies with too few participants (<30 participants) would lead to a major increase in the width of the corrected confidence intervals due to having to incorporate the imprecision in the estimated correction factor.

### Generalizability of the calibration study information under the assumption of random within-person measurement error

A reproducible instrument will give consistent answers with repeat administration, while a valid instrument measures what it was designed to measure. A valid instrument is reproducible, but a reproducible instrument is not necessarily valid. Information from calibration studies designed to compare a new instrument with a gold-standard or a reference measure (i.e. to assess ‘relative validity’) usually are not considered to be generalizable because an FFQ developed for one population cannot readily be used in another population, as different groups of people eat different foods. As such, FFQs tend to be developed and assessed for ‘relative validity’ within specific populations. Indeed, many of the calibration studies designed to correct for measurement error described in this systematic review were based on the selection of a subsample of individuals from the main cohort study. In epidemiological studies in which there is a need to correct for measurement error, but in which no calibration study data are available, investigators could incorporate information from an external study provided that there is transportability. Transportability is satisfied when the measurement error model in the external calibration study can reasonably be assumed to be the same as the one that generated the cruder dietary assessment measurement (e.g. FFQ) in the main study [86]. In this situation, the measurement error model parameters estimated can be used for measurement error correction [6, 37, 87]. However, correction factors derived from linear or nonlinear regression calibration should include information from all relevant covariates in the disease model, whilst bearing in mind that the calibration study should have sufficient participants to be able to accommodate all covariates and thus reduce the potential for model over fitting [88]. Buonaccorsi et al. investigated the impact of possible transportability problems (such as the assumed measurement error model is incorrect or the population involved in the calibration study differs from those participants in the main study) and found that this could result in over- or under-estimation of the correction factor in an unpredictable manner [89].

Rather than try to apply the correction factor to the data directly, Caroll et al. [47] have suggested that it is more sensible to extract from the second study the measurement error variance itself, and use this in conjunction with the distribution of mis-measured exposure in the study needing correction for the effects of measurement error [47]. Geelen et al. [34] have recommended that if the research goal is to compare results between studies or populations, calibration to a reference method that performs similarly in different populations, e.g. a 24-h recall, is preferred. Even if the chosen superior dietary instrument does not fulfil all the criteria for a valid reference method, comparability between the associations obtained from different populations would be improved. They argue that this is more relevant for the generalizability of study results, and is a crucial argument for designing a calibration study to supplement dietary information gathered from large epidemiological studies, even with an imperfect reference instrument [34].

### The most robust approach to correct point and interval estimates for measurement error

Linear regression calibration is the most popular and well understood method to correct associations for measurement error in the nutritional literature, and its application is usually (in the case of the assumption of random error) supported by the collection of repeated measurements of the dietary instrument of interest in a sub-sample of individuals from the main epidemiological study. If systematic error is assumed then dietary intake will also need to be measured on a sub-sample using a reference instrument (which may be imperfect). However, we caution that the key assumptions of this approach must be satisfied. This also means that all important covariates should be included in the linear regression calibration model (whilst guarding against over fitting), in order to avoid bias. Second, linear regression calibration requires that the measures obtained by chosen reference instruments (e.g. dietary records) be linearly related to those obtained by the dietary instrument of interest (e.g. FFQ-reported intakes), and that the residuals of these regressions have constant variance. Often it is necessary to apply a transformation (such as a natural logarithm) to the dietary intake variables in order to meet these conditions. Third, linear regression calibration that is based on an imperfect reference instrument (such as 24-h recalls), should always be supplemented by sensitivity analyses (see Table 10) in order to assess and quantify the magnitude of bias that may result from the correlation between the imperfect reference instrument and the FFQ. Fourth, the assessment of episodically consumed foods and the use of concentration biomarkers (or other imperfect reference methods) that are correlated with dietary intakes also require detailed sensitivity analyses in order to assess the effects on diet-disease associations.

**Table 10:** Bias/sensitivity analyses for measurement error correction

A. When should measurement error bias/sensitivity analyses be conducted?
1. When assessment of the observed diet-disease associations was estimated using a crude instrument of dietary intake such as a FFQ.
2. Essential when the study report aims to translate their findings into policy decision-making actions for a variety of stakeholders.
B. How does one select a method to conduct a model measurement error bias/sensitivity analysis?
1. Aim to balance realistic modelling with practicality of conducting the modelling (e.g. availability of software).
2. Report the measurement error bias/sensitivity analyses as transparently as possible, giving clear details of what was done and the assumptions made.
3. Make the statistical analysis code used to conduct these measurement error bias/sensitivity analyses available either as supplementary web material or by publishing it as an appendix to the main report.
C. How does one assign values to the parameters of the model?
1. Assign values based on the latest information from available data such as internal calibration sub-studies or external calibration studies with a similar design.
2. Choose a range of plausible values in order to assess the impact on the overall findings of a range of scenarios.
3. Evaluate the impact of departures from the assumptions of the classical measurement error model (such as correlated errors between the dietary instruments used or non-differential measurement error).
D. How does one present and interpret the measurement error bias/sensitivity analysis?
1. Present the results in the form of a table or figure where it is possible for the reader to see the complete set of analyses performed.
2. Quantify the direction of the bias based on departures from the classical measurement error model on the overall study findings (e.g. are the observed diet-disease associations likely to be over- estimated or under-estimated?).
3. Describe the implications in light of the measurement error bias/sensitivity analysis (are the policy decisions changed or toned-down in light of these findings?).

Biased correction factors and therefore biased corrections can occur when the errors in true and measured exposure are correlated. For example, if the same erroneous database is used to calculate nutrient intakes for the reference instrument and the dietary instrument of interest, the regression coefficient relating the two methods could be overstated. Willet has suggested that although the potential for correlations in errors between dietary instruments used for regression calibration should always be considered, and should be assessed whenever possible, even moderate correlations in these errors do not appear (on the evidence of methodological studies using simulation) to result in much bias if they are ignored when using standard regression calibration correction procedures [18]. The Strengthening Analytical Thinking for Observational Studies (STRATOS) initiative is developing guidance documents on a variety of topics (including addressing measurement error) to help the research community at large to conduct appropriate statistical analyses of observational studies [90].

### Software availability

There are several implementations of regression calibration using SAS software, including user-friendly publicly available implementations (with documentation) of standard regression calibration [37, 42] at [https://www.hsph.harvard.edu/donna-spiegelman/software/blinplus-macro/ (accessed 8 June 2017)] and MVRC [42, 45] at [https://www.hsph.harvard.edu/donna-spiegelman/software/relibpls8/] (accessed 8 June 2017). There is also software that implements the method of Liao [91] for the Cox model that addresses the rare disease assumption, available at [https://www.hsph.harvard.edu/donna-spiegelman/software/rrc-macro/ (accessed 8 June 2017)]. The National Cancer Institute has produced several SAS macros that can be used to analyze data on regular and episodically consumed foods [https://epi.grants.cancer.gov/diet/usualintakes/macros_single.html (accessed 8 June 2017)]. These SAS macros are accompanied by a user guide and other documentation, as well as example datasets and output.

With regard to other software for implementing these approaches, the standard regression calibration approach, the multivariable regression calibration approach, and the simulation extrapolation approach (SIMEX) are all available as part of the *merror* routines in Stata [92] and there is also a *merror* package in R statistical software [93]. For some of the non-standard approaches, the authors provide statistical code for the implementation for their methods in the original publications (e.g. structural equation modelling (64) and auxiliary information regression calibration [59]). Pérez et al. [94] provide details of an R function that can implement an extension to the two-part method of Kipnis et al. [59] for episodically consumed foods into a three-part method that also incorporates the estimation of amount of energy intake per day. In addition Stata [92], SAS [95], and R [96] have the capability to conduct user defined structural equation modelling analyses and imputation methods.

## Conclusions

The findings of this systematic review provide insights into how to perform calibration studies to quantify and correct for measurement error. Calibration studies of sufficient size and representative of the main study can be very useful in assessing the ‘relative validity’ of dietary instruments as well as the measurement error structure of the dietary instrument being used in the main study. While previous reviews of measurement error correction methods have concentrated on the more technical statistical details of the methods, [97–102], our aim was to review the approaches used to quantify or correct for measurement error in the context of their application to a continuous dietary exposure measurement for a nutritional epidemiological audience. As a consequence, we may not have included some methods that have been described elsewhere if they were not applied to nutritional data. Regression calibration is the most widely used approach to correct for measurement error in nutritional epidemiology; however, it is crucial to ensure that the regression calibration assumptions are fully met. We have discussed other methods proposed to address situations in which the assumptions and requirements of regression calibration are not met. The choice of method should depend on: a) what measurement error model is assumed; b) whether the correct type of calibration study data are available for valid and appropriate correction; and c) if there is potential for bias due to violation of the classical measurement error model assumptions. We propose that sensitivity analyses be conducted in order to assess the impact on diet-disease associations of departures from the classical measurement error model assumptions, and that these be documented and reported in published manuscripts.

## Additional files

Additional file 1: Table S1. Reports that described the development of a method to quantify, or correct for measurement error (RTF 268 kb)

Additional file 2: Table S2. Reports that applied an existing method to quantify, or correct for measurement error (RTF 578 kb)

## Abbreviations

AIRC: Auxiliary information regression calibration
BAGSRC: Biomarker and alloyed gold standard regression calibration
ECFRC: Episodically consumed foods regression calibration
FFQ: Food frequency questionnaire
FRIRC: Flawed reference instrument regression calibration
ICC: Intra class correlation
MI: Multiple imputation
MOTEX1: Method of triads extension 1
MOTEX2: Method of triads extension 2
MOTEX3: Method of triads extension 3
MR: Moment reconstruction
MVRC: Multivariable regression calibration
NEC: Never or episodic consumers
PSBRC: Person specific bias adjusted regression calibration
SIMEX: Simulation extrapolation
STRATOS: Strengthening analytical thinking for observational studies
