# Supplementary material for: Systematic review of statistical approaches to quantify, or correct for, measurement error in a continuous exposure in nutritional epidemiology
Source: BMC Med Res Methodol. 2017 Sep 19;17:146. doi: 10.1186/s12874-017-0421-6 (PMC5606038; doi:10.1186/s12874-017-0421-6)
Supplement: Supplementary file 1 — Reports that described the development of a method to quantify, or correct for measurement error (RTF 268 kb) [file 12874_2017_421_MOESM1_ESM.rtf]

Additonal file 1: Table S1: Reports that described the development of a method to quantify, or correct for measurement error

Lead or
corresponding
author	Main methods developed and described	
Ferrari (2008b)(1)	Bayesian hierarchical model	
Johnson(2)	Bayesian structural measurement model	
Rosner (1989)(3)	Classical Regression Calibration	
Rosner (1990)(4)	Classical Regression Calibration or Variants	
Rosner (1992)(5)	Classical Regression Calibration or Variants	
Spiegelman(6)	Classical Regression Calibration or Variants	
Ferrari (2008a)(7)	Classical Regression Calibration or Variants	
Freedman(2008)(8)	Classical Regression Calibration or Variants/Moment reconstruction/Imputation	
Kipnis (2003)(9)	Classical Regression Calibration or Variants	
Beydoun(10)	Classical Regression Calibration or Variants/SIMEX/Structural Equation Modelling	
Ferrari(2009)(11)	Classical Regression Calibration or Variants	
Fraser (2001)(12)	Classical Regression Calibration or Variants	
Kipnis (2009)(13)	Classical Regression Calibration or Variants	
Kipnis (2001)(14)	Classical Regression Calibration or Variants	
Rosner (2008)(15)	Classical Regression Calibration or Variants	
Suzuki(16)	Classical Regression Calibration or Variants	
Agogo (2016)(17)	Classical Regression Calibration or Variants	
Keogh (2013)(18)	Classical Regression Calibration or Variants	
Yi (19)	Classical Regression Calibration or Variants/SIMIEX/Structural Equation Modelling	
Kipnis (2016)(20)	Classical Regression Calibration or Variants	
Buanocorssi (21)	Classical Regression Calibration or Variants	
Khudyakov (22)	Classical Regression Calibration or Variants	
Nawarantha(23)	Classical Regression Calibration or Variants	
Rosner (2015)(24)	Classical Regression Calibration or Variants/Method of triads	
Freedman (2004)(25)	Classical Regression Calibration or Variants/Correlation	
Keogh (2014)(26)	Classical Regression Calibration or Variants/Moment reconstruction/Imputation	
Prentice (2013)(27)	Classical Regression Calibration or Variants	
Mossavar-Rahmani (2015)(28)	Classical Regression Calibration or Variants	
Pérez(29)	Classical Regression Calibration or Variants	
Fraser (2012)(30)	Classical Regression Calibration or Variants	
Shaw(31) 	Classical Regression Calibration or Variants	
Freedman (2011)(32)	Classical Regression Calibration or Variants	
Liao(33)	Classical Regression Calibration or Variants	
Rosner (1992)(5)	Classical Regression Calibration or Variants	
Daures(34)	Correlation	
Day(35)	Correlation	
Subar(36)	Correlation/ Classical Regression Calibration or Variants	
Orfanos(37)	Correlation/ Classical Regression Calibration or Variants	
Fraser (2004)(38)	Estimating equations	
Fraser (2005)(39)	Method of triads	
Ocke(40)	Method of triads	
Geelen(41)	Method of triads	
Ferrari (2004)(42)	Multi-level model Intra-class correlation	
Potgeiter (2016)(43)	Moment reconstruction/Imputation	


REFERENCES

1.	Ferrari P, Carroll RJ, Gustafson P, Riboli E. A Bayesian multilevel model for estimating the diet/disease relationship in a multicenter study with exposures measured with error: the EPIC study. Statistics in medicine. 2008;27(29):6037-54.
2.	Johnson BA, Herring AH, Ibrahim JG, Siega-Riz AM. Structured measurement error in nutritional epidemiology: applications in the Pregnancy, Infection, and Nutrition (PIN) Study. Journal of the American Statistical Association. 2007;102(479):856-66.
3.	Rosner B, Willett WC, Spiegelman D. Correction of logistic regression relative risk estimates and confidence intervals for systematic within-person measurement error. Statistics in medicine. 1989;8(9):1051-69; discussion 71-3.
4.	Rosner B, Spiegelman D, Willett WC. Correction of logistic regression relative risk estimates and confidence intervals for measurement error: the case of multiple covariates measured with error. American journal of epidemiology. 1990;132(4):734-45. Epub 1990/10/01.
5.	Rosner B, Spiegelman D, Willett WC. Correction of logistic regression relative risk estimates and confidence intervals for random within-person measurement error. American journal of epidemiology. 1992;136(11):1400-13.
6.	Spiegelman D, Schneeweiss S, McDermott A. Measurement error correction for logistic regression models with an "alloyed gold standard". American journal of epidemiology. 1997;145(2):184-96.
7.	Ferrari P, Day NE, Boshuizen HC, Roddam A, Hoffmann K, Thiebaut A, et al. The evaluation of the diet/disease relation in the EPIC study: considerations for the calibration and the disease models. Int J Epidemiol. 2008;37(2):368-78.
8.	Freedman LS, Midthune D, Carroll RJ, Kipnis V. A comparison of regression calibration, moment reconstruction and imputation for  adjusting for covariate measurement error in regression. Statistics in medicine. 2008;27(25):5195-216.
9.	Kipnis V, Subar AF, Midthune D, Freedman LS, Ballard-Barbash R, Troiano RP, et al. Structure of dietary measurement error: results of the OPEN biomarker study. American journal of epidemiology. 2003;158(1):14-21; discussion 2-6.
10.	Beydoun MA, Kaufman JS, Ibrahim J, Satia JA, Heiss G. Measurement error adjustment in essential fatty acid intake from a food frequency questionnaire: alternative approaches and methods. BMC Med Res Methodol. 2007;7:41.
11.	Ferrari P, Roddam A, Fahey MT, Jenab M, Bamia C, Ocke M, et al. A bivariate measurement error model for nitrogen and potassium intakes to evaluate the performance of regression calibration in the European Prospective Investigation into Cancer and Nutrition study. European journal of clinical nutrition. 2009;63 Suppl 4:S179-87.
12.	Fraser GE, Stram DO. Regression calibration in studies with correlated variables measured with error. American journal of epidemiology. 2001;154(9):836-44.
13.	Kipnis V, Midthune D, Buckman DW, Dodd KW, Guenther PM, Krebs-Smith SM, et al. Modeling data with excess zeros and measurement error: application to evaluating  relationships between episodically consumed foods and health outcomes. Biometrics. 2009;65(4):1003-10.
14.	Kipnis V, Midthune D, Freedman LS, Bingham S, Schatzkin A, Subar A, et al. Empirical evidence of correlated biases in dietary assessment instruments and its implications. American journal of epidemiology. 2001;153(4):394-403.
15.	Rosner B, Michels KB, Chen YH, Day NE. Measurement error correction for nutritional exposures with correlated measurement error: use of the method of triads in a longitudinal setting. Statistics in medicine. 2008;27(18):3466-89.
16.	Suzuki R, Allen NE, Key TJ, Appleby PN, Tjonneland A, Johnsen NF, et al. A prospective analysis of the association between dietary fiber intake and prostate cancer risk in EPIC. Int J Cancer. 2009;124(1):245-9.
17.	Agogo GO, van der Voet H, van't Veer P, van Eeuwijk FA, Boshuizen HC. Evaluation of a two-part regression calibration to adjust for dietary exposure measurement error in the Cox proportional hazards model: A simulation study. Biometrical Journal. 2016:n/a-n/a.
18.	Keogh RH, White IR, Rodwell SA. Using surrogate biomarkers to improve measurement error models in nutritional epidemiology. Statistics in medicine. 2013;32(22):3838-61. Epub 2013/04/05.
19.	Yi GY, Ma Y, Spiegelman D, Carroll RJ. Functional and structural methods with mixed measurement error and misclassification in covariates. Journal of the American Statistical Association. 2015;110(510):681-96.
20.	Kipnis V, Freedman LS, Carroll RJ, Midthune D. A bivariate measurement error model for semicontinuous and continuous variables: Application to nutritional epidemiology. Biometrics. 2016;72(1):106-15.
21.	Buonaccorsi JP, Dalen I, Laake P, Hjartåker A, Engeset D, Thoresen M. Sensitivity of regression calibration to non-perfect validation data with application to the Norwegian Women and Cancer Study. Statistics in medicine. 2015;34(8):1389-403.
22.	Khudyakov P, Gorfine M, Zucker D, Spiegelman D. The Impact of Covariate Measurement Error on Risk Prediction. Statistics in medicine. 2015;34(15):2353-67.
23.	Nawarathna LS, Choudhary PK. A heteroscedastic measurement error model for method comparison data with replicate measurements. Statistics in medicine. 2015;34(7):1242-58.
24.	Rosner B, Hendrickson S, Willett W. Optimal Allocation of Resources in a Biomarker Setting. Statistics in medicine. 2015;34(2):297-306.
25.	Freedman LS, Fainberg V, Kipnis V, Midthune D, Carroll RJ. A new method for dealing with measurement error in explanatory variables of regression models. Biometrics. 2004;60(1):172-81.
26.	Keogh RH, White IR. A toolkit for measurement error correction, with a focus on nutritional epidemiology. Statistics in medicine. 2014;33(12):2137-55. Epub 2014/02/06.
27.	Prentice RL, Tinker LF, Huang Y, Neuhouser ML. Calibration Of Self-Reported Dietary Measures Using Biomarkers: An Approach To Enhancing Nutritional Epidemiology Reliability. Current atherosclerosis reports. 2013;15(9):10.1007/s11883-013-0353-5.
28.	Mossavar-Rahmani Y, Shaw PA, Wong WW, Sotres-Alvarez D, Gellman MD, Van Horn L, et al. Applying Recovery Biomarkers to Calibrate Self-Report Measures of Energy and Protein in the Hispanic Community Health Study/Study of Latinos. American journal of epidemiology. 2015;181(12):996-1007.
29.	Perez A, Zhang S, Kipnis V, Midthune D, Freedman LS, Carroll RJ. Intake_epis_food(): An R Function for Fitting a Bivariate Nonlinear Measurement Error Model to Estimate Usual and Energy Intake for Episodically Consumed Foods. Journal of statistical software. 2012;46(c03):1-17. Epub 2012/07/28.
30.	Fraser GE, Stram DO. Regression calibration when foods (measured with error) are the variables of interest: markedly non-Gaussian data with many zeroes. American journal of epidemiology. 2012;175(4):325-31.
31.	Shaw PA, Prentice RL. Hazard ratio estimation for biomarker-calibrated dietary exposures. Biometrics. 2012;68(2):397-407.
32.	Freedman LS, Midthune D, Carroll RJ, Tasevska N, Schatzkin A, Mares J, et al. Using regression calibration equations that combine self-reported intake and biomarker measures to obtain unbiased estimates and more powerful tests of dietary associations. American journal of epidemiology. 2011;174(11):1238-45. Epub 2011/11/04.
33.	Liao X, Zucker DM, Li Y, Spiegelman D. Survival analysis with error-prone time-varying covariates: a risk set calibration approach. Biometrics. 2011;67(1):50-8. Epub 2010/05/22.
34.	Daures JP, Gerber M, Scali J, Astre C, Bonifacj C, Kaaks R. Validation of a food-frequency questionnaire using multiple-day records and biochemical markers: application of the triads method. J Epidemiol Biostat. 2000;5(2):109-15.
35.	Day NE, Wong MY, Bingham S, Khaw KT, Luben R, Michels KB, et al. Correlated measurement error--implications for nutritional epidemiology. Int J Epidemiol. 2004;33(6):1373-81.
36.	Subar AF, Kipnis V, Troiano RP, Midthune D, Schoeller DA, Bingham S, et al. Using intake biomarkers to evaluate the extent of dietary misreporting in a large sample of adults: the OPEN study. American journal of epidemiology. 2003;158(1):1-13. Epub 2003/07/02.
37.	Orfanos P, Knüppel S, Naska A, Haubrock J, Trichopoulou A, Boeing H. Evaluating the effect of measurement error when using one or two 24 h dietary recalls to assess eating out: a study in the context of the HECTOR project. Brit J Nutr. 2013;110(6):1107-17.
38.	Fraser GE, Shavlik DJ. Correlations between estimated and true dietary intakes. Annals of epidemiology. 2004;14(4):287-95.
39.	Fraser GE, Butler TL, Shavlik D. Correlations between estimated and true dietary intakes: using two instrumental variables. Annals of epidemiology. 2005;15(7):509-18.
40.	Ocke MC, Kaaks RJ. Biochemical markers as additional measurements in dietary validity studies: application of the method of triads with examples from the European Prospective Investigation into Cancer and Nutrition. The American journal of clinical nutrition. 1997;65(4 Suppl):1240S-5S.
41.	Geelen A, Souverein OW, Busstra MC, de Vries JH, van 't Veer P. Comparison of approaches to correct intake-health associations for FFQ measurement error using a duplicate recovery biomarker and a duplicate 24 h dietary recall as reference method. Public Health Nutr. 2014:1-8. Epub 2014/02/07.
42.	Ferrari P, Kaaks R, Fahey MT, Slimani N, Day NE, Pera G, et al. Within- and between-cohort variation in measured macronutrient intakes, taking account of measurement errors, in the European Prospective Investigation into Cancer and Nutrition study. American journal of epidemiology. 2004;160(8):814-22.
43.	Potgieter CJ, Wei R, Kipnis V, Freedman LS, Carroll RJ. Moment reconstruction and moment-adjusted imputation when exposure is generated by a complex, nonlinear random effects modeling process. Biometrics. 2016;72(4):1369-77.
