# Supplementary material for: Systematic review of statistical approaches to quantify, or correct for, measurement error in a continuous exposure in nutritional epidemiology
Source: BMC Med Res Methodol. 2017 Sep 19;17:146. doi: 10.1186/s12874-017-0421-6 (PMC5606038; doi:10.1186/s12874-017-0421-6)
Supplement: Supplementary file 2 — Reports that applied an existing method to quantify, or correct for measurement error (RTF 578 kb) [file 12874_2017_421_MOESM2_ESM.rtf]

Additional file 2: Table S2: Reports that applied an existing method to quantify, or correct for measurement error

Lead or
corresponding
author	Main method
applied	
Dahm(1)	Classical Regression Calibration or Variants	
Buckland(2)	Classical Regression Calibration or Variants	
Buchner(3)	Classical Regression Calibration or Variants	
Thompson(4)	Classical Regression Calibration or Variants	
Cust(5)	Classical Regression Calibration or Variants	
Linseisen (2007)(6)	Classical Regression Calibration or Variants	
Cade(7)	Classical Regression Calibration or Variants	
Thiebaut(8)	Classical Regression Calibration or Variants	
Parr (2006)(9)	Classical Regression Calibration or Variants	
Schatzkin(10)	Classical Regression Calibration or Variants	
Slimani(11)	Classical Regression Calibration or Variants	
Johannson(12)	Classical Regression Calibration or Variants	
Kynast-Wolfe(13)	Classical Regression Calibration or Variants	
Beasley(14)	Classical Regression Calibration or Variants	
Prentice(15)	Classical Regression Calibration or Variants	
Horn-Ross (2008)(16)	Classical Regression Calibration or Variants /Correlations	
Gonzalez(17)	Classical Regression Calibration or Variants	
Boeing(18)	Classical Regression Calibration or Variants	
Hunter(19)	Classical Regression Calibration or Variants	
Allen (2008) (20)	Classical Regression Calibration or Variants	
Bingham(21)	Classical Regression Calibration or Variants	
Agogo (2014)(22)	Classical Regression Calibration or Variants	
Tasaveska (2014)(23)	Classical Regression Calibration or Variants	
Jaacks (24)	Classical Regression Calibration or Variants	
Huang (25)	Classical Regression Calibration or Variants	
Zhang , Z (2013)(26)	Classical Regression Calibration or Variants	
Rohrmann(27)	Classical Regression Calibration or Variants	
Parr (2013)(28)	Classical Regression Calibration or Variants	
Keogh (2012)(29)	Classical Regression Calibration or Variants	
Deziel (30)	Classical Regression Calibration or Variants	
Tinker (31))	Classical Regression Calibration or Variants	
Zhang, X (2011)(32)	Classical Regression Calibration or Variants	
Midthune (2011)(33)	Classical Regression Calibration or Variants	
Linseisen (2011)(34)	Classical Regression Calibration or Variants	
Keogh (2011)(35)	Classical Regression Calibration or Variants	
Tasaveska (2011)(36)	Classical Regression Calibration or Variants	
Liese (2015a)(37)	Classical Regression Calibration or Variants	
Zhang , Z (2015)(38)	Classical Regression Calibration or Variants	
Mossavar-Rahmani (2013(39))	Classical Regression Calibration or Variants	
Brantsaeter(2009)(40)	Classical Regression Calibration or Variants	
Frobisher(41)	Classical Regression Calibration or Variants	
Jaceldo-Siegl (2010)(42)	Correlation	
Jaceldo-Siegl (2008)(43)	Correlation	
Rautainen(44)	Correlation	
Pasanisi(45)	Correlation	
Kroke(46)	Correlation	
Shimizu(47)	Correlation	
Klipstein-Grobusch(48)	Correlation	
Du(49)	Correlation	
Horn-Ross (2006)(50)	Intra-class correlation	
Carithers(51)	Correlation	
Katsouyanni(52)	Correlation	
MacInytre(53)	Correlation	
Zhang, B (2010)(54)	Correlation	
Lassale (2015)(55)	Correlation	
Joh (2015)(56) 	Correlation	
Mercado (2015)(57)	Correlation	
Cross (2014)(58)	Correlation	
Liese (2015b)(59) 	Correlation	
Song (2014)(60)	Correlation	
Feskens (2013)(61)	Correlation	
Allen (2012)(62)	Correlation	
Arab (2011)(63)	Correlation	
Lassale (2016)(64)	Correlation	
Liese (2014)(65)	Correlation	
Brantsaeter (2007)(66)	Correlation	
Andersen(67)	Correlation	
Bhakta(68)	Correlation	
Bidulescu (2009)(69)	Correlation	
Bidulescu (2007)(70)	Correlation	
Day (2001)(71)	Correlation	
Dixon(72)	Method of triads	
Fowke(73)	Method of triads	
Kabagambe(74)	Method of triads	
McNaughton (2007)(75)	Method of triads	
McNaughton (2005)(76)	Method of triads	
Shai(77)	Method of triads	
Ollberding (2015)(78)	Intra-class correlation/Mixed-effect linear regression models	
Ferrari (2005)(79)	Multi-level model /Intra-class correlation	
Polusna(80)	Not reported	
Kirkpatrick (2014)(81)	Not reported	
Dyer(82)	One-way random effects ANOVA/Intra-class correlation	
Börnhorst (2013)(83)	Coefficient of variation	


REFERENCES
1.	Dahm CC, Keogh RH, Lentjes MA, Spencer EA, Key TJ, Greenwood DC, et al. Intake of dietary fats and colorectal cancer risk: prospective findings from the  UK Dietary Cohort Consortium. Cancer epidemiology. 2010;34(5):562-7.
2.	Buckland G, Agudo A, Lujan L, Jakszyn P, Bueno-de-Mesquita HB, Palli D, et al. Adherence to a Mediterranean diet and risk of gastric adenocarcinoma within the European Prospective Investigation into Cancer and Nutrition (EPIC) cohort study. The American journal of clinical nutrition. 2010;91(2):381-90.
3.	Buchner FL, Bueno-de-Mesquita HB, Ros MM, Kampman E, Egevad L, Overvad K, et al. Consumption of vegetables and fruit and the risk of bladder cancer in the European Prospective Investigation into Cancer and Nutrition. Int J Cancer. 2009;125(11):2643-51.
4.	Thompson FE, Kipnis V, Midthune D, Freedman LS, Carroll RJ, Subar AF, et al. Performance of a food-frequency questionnaire in the US NIH-AARP (National Institutes of Health-American Association of Retired Persons) Diet and Health Study. Public Health Nutr. 2008;11(2):183-95.
5.	Cust AE, Slimani N, Kaaks R, van Bakel M, Biessy C, Ferrari P, et al. Dietary carbohydrates, glycemic index, glycemic load, and endometrial cancer risk within the European Prospective Investigation into Cancer and Nutrition cohort. American journal of epidemiology. 2007;166(8):912-23.
6.	Linseisen J, Rohrmann S, Miller AB, Bueno-de-Mesquita HB, Buchner FL, Vineis P, et al. Fruit and vegetable consumption and lung cancer risk: updated information from the European Prospective Investigation into Cancer and Nutrition (EPIC). Int J Cancer. 2007;121(5):1103-14.
7.	Cade JE, Burley VJ, Greenwood DC. Dietary fibre and risk of breast cancer in the UK Women's Cohort Study. Int J Epidemiol. 2007;36(2):431-8.
8.	Thiebaut AC, Kipnis V, Chang SC, Subar AF, Thompson FE, Rosenberg PS, et al. Dietary fat and postmenopausal invasive breast cancer in the National Institutes of Health-AARP Diet and Health Study cohort. Journal of the National Cancer Institute. 2007;99(6):451-62. Epub 2007/03/22.
9.	Parr CL, Veierod MB, Laake P, Lund E, Hjartaker A. Test-retest reproducibility of a food frequency questionnaire (FFQ) and estimated effects on disease risk in the Norwegian Women and Cancer Study (NOWAC). Nutrition journal. 2006;5:4.
10.	Schatzkin A, Kipnis V, Carroll RJ, Midthune D, Subar AF, Bingham S, et al. A comparison of a food frequency questionnaire with a 24-hour recall for use in an epidemiological cohort study: results from the biomarker-based Observing Protein and Energy Nutrition (OPEN) study. Int J Epidemiol. 2003;32(6):1054-62.
11.	Slimani N, Bingham S, Runswick S, Ferrari P, Day NE, Welch AA, et al. Group level validation of protein intakes estimated by 24-hour diet recall and dietary questionnaires against 24-hour urinary nitrogen in the European Prospective Investigation into Cancer and Nutrition (EPIC) calibration study. Cancer epidemiology, biomarkers & prevention : a publication of the American Association for Cancer Research, cosponsored by the American Society of Preventive Oncology. 2003;12(8):784-95.
12.	Johansson I, Hallmans G, Wikman A, Biessy C, Riboli E, Kaaks R. Validation and calibration of food-frequency questionnaire measurements in the Northern Sweden Health and Disease cohort. Public Health Nutr. 2002;5(3):487-96.
13.	Kynast-Wolf G, Becker N, Kroke A, Brandstetter BR, Wahrendorf J, Boeing H. Linear regression calibration: theoretical framework and empirical results in EPIC, Germany. Annals of nutrition & metabolism. 2002;46(1):2-8.
14.	Beasley JM, LaCroix AZ, Neuhouser ML, Huang Y, Tinker L, Woods N, et al. Protein intake and incident frailty in the Women's Health Initiative observational study. Journal of the American Geriatrics Society. 2010;58(6):1063-71.
15.	Prentice RL, Huang Y, Kuller LH, Tinker LF, Horn LV, Stefanick ML, et al. Biomarker-calibrated energy and protein consumption and cardiovascular disease risk among postmenopausal women. Epidemiology. 2011;22(2):170-9.
16.	Horn-Ross PL, Lee VS, Collins CN, Stewart SL, Canchola AJ, Lee MM, et al. Dietary assessment in the California Teachers Study: reproducibility and validity. Cancer causes & control : CCC. 2008;19(6):595-603.
17.	Gonzalez CA, Pera G, Agudo A, Bueno-de-Mesquita HB, Ceroti M, Boeing H, et al. Fruit and vegetable intake and the risk of stomach and oesophagus adenocarcinoma  in the European Prospective Investigation into Cancer and Nutrition (EPIC-EURGAST). Int J Cancer. 2006;118(10):2559-66.
18.	Boeing H, Bohlscheid-Thomas S, Voss S, Schneeweiss S, Wahrendorf J. The relative validity of vitamin intakes derived from a food frequency questionnaire compared to 24-hour recalls and biological measurements: results from the EPIC pilot study in Germany. European Prospective Investigation into Cancer and Nutrition. Int J Epidemiol. 1997;26 Suppl 1:S82-90.
19.	Hunter DJ, Spiegelman D, Adami HO, Beeson L, van den Brandt PA, Folsom AR, et al. Cohort studies of fat intake and the risk of breast cancer--a pooled analysis. The New England journal of medicine. 1996;334(6):356-61.
20.	Allen NE, Key TJ, Appleby PN, Travis RC, Roddam AW, Tjonneland A, et al. Animal foods, protein, calcium and prostate cancer risk: the European Prospective Investigation into Cancer and Nutrition. British journal of cancer. 2008;98(9):1574-81.
21.	Bingham SA, Day NE, Luben R, Ferrari P, Slimani N, Norat T, et al. Dietary fibre in food and protection against colorectal cancer in the European Prospective Investigation into Cancer and Nutrition (EPIC): an observational study. Lancet. 2003;361(9368):1496-501.
22.	Agogo GO, van der Voet H, van't Veer P, Ferrari P, Leenders M, Muller DC, et al. Use of two-part regression calibration model to correct for measurement error in episodically consumed foods in a single-replicate study design: EPIC case study. PloS one. 2014;9(11):e113160. Epub 2014/11/18.
23.	Tasevska N, Midthune D, Tinker LF, Potischman N, Lampe JW, Neuhouser ML, et al. Use of a urinary sugars biomarker to assess measurement error in self-reported sugars intake in the nutrition and physical activity assessment study (NPAAS). Cancer epidemiology, biomarkers & prevention : a publication of the American Association for Cancer Research, cosponsored by the American Society of Preventive Oncology. 2014;23(12):2874-83. Epub 2014/09/23.
24.	Jaacks LM, Crandell J, Liese AD, Lamichhane AP, Bell RA, Dabelea D, et al. No association of dietary fiber intake with inflammation or arterial stiffness in youth with type 1 diabetes. Journal of diabetes and its complications. 2014;28(3):305-10. Epub 2014/03/13.
25.	Huang Y, Van Horn L, Tinker LF, Neuhouser ML, Carbone L, Mossavar-Rahmani Y, et al. Measurement error corrected sodium and potassium intake estimation using 24-hour urinary excretion. Hypertension. 2014;63(2):238-44. Epub 2013/11/28.
26.	Zhang Z, Cogswell ME, Gillespie C, Fang J, Loustalot F, Dai S, et al. Association between usual sodium and potassium intake and blood pressure and hypertension among U.S. adults: NHANES 2005-2010. PloS one. 2013;8(10):e75289. Epub 2013/10/17.
27.	Rohrmann S, Linseisen J, Nothlings U, Overvad K, Egeberg R, Tjonneland A, et al. Meat and fish consumption and risk of pancreatic cancer: results from the European Prospective Investigation into Cancer and Nutrition. Int J Cancer. 2013;132(3):617-24. Epub 2012/05/23.
28.	Parr CL, Hjartaker A, Lund E, Veierod MB. Meat intake, cooking methods and risk of proximal colon, distal colon and rectal cancer: the Norwegian Women and Cancer (NOWAC) cohort study. Int J Cancer. 2013;133(5):1153-63. Epub 2013/02/13.
29.	Keogh RH, Park JY, White IR, Lentjes MA, McTaggart A, Bhaniani A, et al. Estimating the alcohol-breast cancer association: a comparison of diet diaries, FFQs and combined measurements. European journal of epidemiology. 2012;27(7):547-59. Epub 2012/05/31.
30.	Deziel NC, Buckley TJ, Sinha R, Abubaker S, Platz EA, Strickland PT. Comparability and repeatability of methods for estimating the dietary intake of the heterocyclic amine contaminant 2-amino-1-methyl-6-phenylimidazo[4,5b]pyridine (PhIP). Food additives & contaminants Part A, Chemistry, analysis, control, exposure & risk assessment. 2012;29(8):1202-11. Epub 2012/05/11.
31.	Tinker LF, Sarto GE, Howard BV, Huang Y, Neuhouser ML, Mossavar-Rahmani Y, et al. Biomarker-calibrated dietary energy and protein intake associations with diabetes risk among postmenopausal women from the Women's Health Initiative. The American journal of clinical nutrition. 2011;94(6):1600-6. Epub 2011/11/11.
32.	Zhang X, Smith-Warner SA, Chan AT, Wu K, Spiegelman D, Fuchs CS, et al. Aspirin use, body mass index, physical activity, plasma C-peptide, and colon cancer risk in US health professionals. American journal of epidemiology. 2011;174(4):459-67. Epub 2011/06/16.
33.	Midthune D, Schatzkin A, Subar AF, Thompson FE, Freedman LS, Carroll RJ, et al. Validating an FFQ for intake of episodically consumed foods: application to the National Institutes of Health-AARP Diet and Health Study. Public Health Nutr. 2011;14(7):1212-21.
34.	Linseisen J, Rohrmann S, Bueno-de-Mesquita B, Buchner FL, Boshuizen HC, Agudo A, et al. Consumption of meat and fish and risk of lung cancer: results from the European Prospective Investigation into Cancer and Nutrition. Cancer causes & control : CCC. 2011;22(6):909-18. Epub 2011/04/12.
35.	Keogh RH, White IR. Allowing for never and episodic consumers when correcting for error in food record measurements of dietary intake. Biostatistics. 2011;12(4):624-36. Epub 2011/03/08.
36.	Tasevska N, Midthune D, Potischman N, Subar AF, Cross AJ, Bingham SA, et al. Use of the predictive sugars biomarker to evaluate self-reported total sugars intake in the Observing Protein and Energy Nutrition (OPEN) study. Cancer epidemiology, biomarkers & prevention : a publication of the American Association for Cancer Research, cosponsored by the American Society of Preventive Oncology. 2011;20(3):490-500. Epub 2011/02/01.
37.	Liese AD, Crandell JL, Tooze JA, Fangman MT, Couch SC, Merchant AT, et al. Relative validity and reliability of an FFQ in youth with type 1 diabetes. Public Health Nutr. 2015;18(3):428-37. Epub 2014/04/01.
38.	Zhang Z, Gillespie C, Welsh JA, Hu FB, Yang Q. Usual Intake of Added Sugars and Lipid Profiles Among the U.S. Adolescents: National Health and Nutrition Examination Survey, 2005–2010. The Journal of adolescent health : official publication of the Society for Adolescent Medicine. 2015;56(3):352-9.
39.	Mossavar-Rahmani Y, Tinker LF, Huang Y, Neuhouser ML, McCann SE, Seguin RA, et al. Factors relating to eating style, social desirability, body image and eating meals at home increase the precision of calibration equations correcting self-report measures of diet using recovery biomarkers: findings from the Women's Health Initiative. Nutrition journal. 2013;12:63. Epub 2013/05/18.
40.	Brantsaeter AL, Haugen M, Julshamn K, Alexander J, Meltzer HM. Evaluation of urinary iodine excretion as a biomarker for intake of milk and dairy products in pregnant women in the Norwegian Mother and Child Cohort Study (MoBa). European journal of clinical nutrition. 2009;63(3):347-54.
41.	Frobisher C, Tilling K, Emmett PM, Maynard M, Ness AR, Davey Smith G, et al. Reproducibility measures and their effect on diet-cancer associations in the Boyd Orr cohort. J Epidemiol Community Health. 2007;61(5):434-40.
42.	Jaceldo-Siegl K, Knutsen SF, Sabate J, Beeson WL, Chan J, Herring RP, et al. Validation of nutrient intake using an FFQ and repeated 24 h recalls in black and white subjects of the Adventist Health Study-2 (AHS-2). Public Health Nutr. 2010;13(6):812-9.
43.	Jackson C, Best N, Elliott P. UK Biobank Pilot Study: stability of haematological and clinical chemistry analytes. Int J Epidemiol. 2008;37 Suppl 1:i16-22.
44.	Rautiainen S, Serafini M, Morgenstern R, Prior RL, Wolk A. The validity and reproducibility of food-frequency questionnaire-based total antioxidant capacity estimates in Swedish women. The American journal of clinical nutrition. 2008;87(5):1247-53.
45.	Pasanisi P, Berrino F, Bellati C, Sieri S, Krogh V. Validity of the Italian EPIC questionnaire to assess past diet. IARC scientific publications. 2002;156:41-4.
46.	Kroke A, Klipstein-Grobusch K, Voss S, Moseneder J, Thielecke F, Noack R, et al. Validation of a self-administered food-frequency questionnaire administered in the European Prospective Investigation into Cancer and Nutrition (EPIC) Study: comparison of energy, protein, and macronutrient intakes estimated with the doubly labeled water, urinary nitrogen, and repeated 24-h dietary recall methods. The American journal of clinical nutrition. 1999;70(4):439-47.
47.	Shimizu H, Ohwaki A, Kurisu Y, Takatsuka N, Ido M, Kawakami N, et al. Validity and reproducibility of a quantitative food frequency questionnaire for a cohort study in Japan. Jpn J Clin Oncol. 1999;29(1):38-44.
48.	Klipstein-Grobusch K, den Breeijen JH, Goldbohm RA, Geleijnse JM, Hofman A, Grobbee DE, et al. Dietary assessment in the elderly: validation of a semiquantitative food frequency questionnaire. European journal of clinical nutrition. 1998;52(8):588-96.
49.	Du H, van der AD, van Bakel MM, Verberne LD, Ocke M, Feskens EJ. Reproducibility and relative validity of dietary glycaemic index and glycaemic load assessed by the food-frequency questionnaire used in the Dutch cohorts of the European Prospective Investigation into Cancer and Nutrition. The British journal of nutrition. 2009;102(4):601-4.
50.	Horn-Ross PL, Barnes S, Lee VS, Collins CN, Reynolds P, Lee MM, et al. Reliability and validity of an assessment of usual phytoestrogen consumption (United States). Cancer causes & control : CCC. 2006;17(1):85-93.
51.	Carithers T, Dubbert PM, Crook E, Davy B, Wyatt SB, Bogle ML, et al. Dietary assessment in African Americans: methods used in the Jackson Heart Study. Ethnicity & disease. 2005;15(4 Suppl 6):S6-49-55.
52.	Katsouyanni K, Rimm EB, Gnardellis C, Trichopoulos D, Polychronopoulos E, Trichopoulou A. Reproducibility and relative validity of an extensive semi-quantitative food frequency questionnaire using dietary records and biochemical markers among Greek schoolteachers. Int J Epidemiol. 1997;26 Suppl 1:S118-27.
53.	MacIntyre UE, Venter CS, Vorster HH. A culture-sensitive quantitative food frequency questionnaire used in an African  population: 2. Relative validation by 7-day weighted records and biomarkers. Public Health Nutr. 2001;4(1):63-71.
54.	Zhang B, Wang P, Chen CG, He QQ, Zhuo SY, Chen YM, et al. Validation of an FFQ to estimate the intake of fatty acids using erythrocyte membrane fatty acids and multiple 3d dietary records. Public Health Nutr. 2010;13(10):1546-52.
55.	Lassale C, Castetbon K, Laporte F, Camilleri GM, Deschamps V, Vernay M, et al. Validation of a Web-based, self-administered, non-consecutive-day dietary record tool against urinary biomarkers. The British journal of nutrition. 2015:1-10. Epub 2015/03/17.
56.	Joh HK, Oh SW, Lee E. Reproducibility and validity of semi-quantitative food frequency questionnaire measuring dietary trans-fatty acids intake among Korean adults. Nutrition research and practice. 2015;9(1):99-105. Epub 2015/02/12.
57.	Mercado CI, Cogswell ME, Valderrama AL, Wang CY, Loria CM, Moshfegh AJ, et al. Difference between 24-h diet recall and urine excretion for assessing population sodium and potassium intake in adults aged 18-39 y. The American journal of clinical nutrition. 2015;101(2):376-86. Epub 2015/02/04.
58.	Cross AJ, Major JM, Rothman N, Sinha R. Urinary 1-methylhistidine and 3-methylhistidine, meat intake, and colorectal adenoma risk. Eur J Cancer Prev. 2014;23(5):385-90. Epub 2014/04/01.
59.	Liese AD, Crandell JL, Tooze JA, Kipnis V, Bell R, Couch SC, et al. Sugar-sweetened beverage intake and cardiovascular risk factor profile in youth with type 1 diabetes: application of measurement error methodology in the SEARCH Nutrition Ancillary Study. Brit J Nutr. 2015;114(3):430-8.
60.	Song X, Navarro SL, Diep P, Thomas WK, Razmpoosh EC, Schwarz Y, et al. Comparison and validation of 2 analytical methods for measurement of urinary sucrose and fructose excretion. Nutr Res. 2013;33(9):696-703. Epub 2013/09/17.
61.	Feskens EJ, Sluik D, van Woudenbergh GJ. Meat consumption, diabetes, and its complications. Current diabetes reports. 2013;13(2):298-306. Epub 2013/01/29.
62.	Allen NE, Appleby PN, Key TJ, Bueno-de-Mesquita HB, Ros MM, Kiemeney LA, et al. Macronutrient intake and risk of urothelial cell carcinoma in the European prospective investigation into cancer and nutrition. Int J Cancer. 2013;132(3):635-44. Epub 2012/05/24.
63.	Arab L, Wesseling-Perry K, Jardack P, Henry J, Winter A. Eight self-administered 24-hour dietary recalls using the Internet are feasible in African Americans and Whites: the energetics study. J Am Diet Assoc. 2010;110(6):857-64.
64.	Lassale C, Castetbon K, Laporte F, Deschamps V, Vernay M, Camilleri GM, et al. Correlations between Fruit, Vegetables, Fish, Vitamins, and Fatty Acids Estimated by Web-Based Nonconsecutive Dietary Records and Respective Biomarkers of Nutritional Status. J Acad Nutr Diet. 2016;116(3):427-38.e5.
65.	Liese AD, Bell BA, Barnes TL, Colabianchi N, Hibbert JD, Blake CE, et al. Environmental influences on fruit and vegetable intake: results from a path analytic model. Public Health Nutr. 2014;17(11):2595-604. Epub 2013/11/07.
66.	Brantsaeter AL, Haugen M, Rasmussen SE, Alexander J, Samuelsen SO, Meltzer HM. Urine flavonoids and plasma carotenoids in the validation of fruit, vegetable and tea intake during pregnancy in the Norwegian Mother and Child Cohort Study (MoBa). Public Health Nutr. 2007;10(8):838-47.
67.	Andersen LF, Veierod MB, Johansson L, Sakhi A, Solvoll K, Drevon CA. Evaluation of three dietary assessment methods and serum biomarkers as measures of fruit and vegetable intake, using the method of triads. The British journal of nutrition. 2005;93(4):519-27.
68.	Bhakta D, dos Santos Silva I, Higgins C, Sevak L, Kassam-Khamis T, Mangtani P, et al. A semiquantitative food frequency questionnaire is a valid indicator of the usual intake of phytoestrogens by south Asian women in the UK relative to multiple 24-h dietary recalls and multiple plasma samples. J Nutr. 2005;135(1):116-23.
69.	Bidulescu A, Chambless LE, Siega-Riz AM, Zeisel SH, Heiss G. Repeatability and measurement error in the assessment of choline and betaine dietary intake: the Atherosclerosis Risk in Communities (ARIC) study. Nutrition journal. 2009;8:14.
70.	Bidulescu A, Chambless LE, Siega-Riz AM, Zeisel SH, Heiss G. Usual choline and betaine dietary intake and incident coronary heart disease: the Atherosclerosis Risk in Communities (ARIC) study. BMC cardiovascular disorders. 2007;7:20.
71.	Day N, McKeown N, Wong M, Welch A, Bingham S. Epidemiological assessment of diet: a comparison of a 7-day diary with a food frequency questionnaire using urinary markers of nitrogen, potassium and sodium. Int J Epidemiol. 2001;30(2):309-17.
72.	Dixon LB, Subar AF, Wideroff L, Thompson FE, Kahle LL, Potischman N. Carotenoid and tocopherol estimates from the NCI diet history questionnaire are valid compared with multiple recalls and serum biomarkers. J Nutr. 2006;136(12):3054-61.
73.	Fowke JH, Hebert JR, Fahey JW. Urinary excretion of dithiocarbamates and self-reported Cruciferous vegetable intake: application of the 'method of triads' to a food-specific biomarker. Public Health Nutr. 2002;5(6):791-9.
74.	Kabagambe EK, Baylin A, Allan DA, Siles X, Spiegelman D, Campos H. Application of the method of triads to evaluate the performance of food frequency questionnaires and biomarkers as indicators of long-term dietary intake. American journal of epidemiology. 2001;154(12):1126-35. Epub 2001/12/18.
75.	McNaughton SA, Hughes MC, Marks GC. Validation of a FFQ to estimate the intake of PUFA using plasma phospholipid fatty acids and weighed foods records. The British journal of nutrition. 2007;97(3):561-8.
76.	McNaughton SA, Marks GC, Gaffney P, Williams G, Green A. Validation of a food-frequency questionnaire assessment of carotenoid and vitamin E intake using weighed food records and plasma biomarkers: the method of triads model. European journal of clinical nutrition. 2005;59(2):211-8.
77.	Shai I, Rosner BA, Shahar DR, Vardi H, Azrad AB, Kanfi A, et al. Dietary evaluation and attenuation of relative risk: multiple comparisons between blood and urinary biomarkers, food frequency, and 24-hour recall questionnaires:  the DEARR study. J Nutr. 2005;135(3):573-9.
78.	Ollberding NJ, Gilsanz V, Lappe JM, Oberfield SE, Shepherd JA, Winer KK, et al. Reproducibility and intermethod reliability of a calcium food frequency questionnaire for use in Hispanic, non-Hispanic black, and non-Hispanic white youth. J Acad Nutr Diet. 2015;115(4):519-27 e2. Epub 2015/02/17.
79.	Ferrari P, Al-Delaimy WK, Slimani N, Boshuizen HC, Roddam A, Orfanos P, et al. An approach to estimate between- and within-group correlation coefficients in multicenter studies: plasma carotenoids as biomarkers of intake of fruits and vegetables. American journal of epidemiology. 2005;162(6):591-8.
80.	Poslusna K, Ruprich J, de Vries JH, Jakubikova M, van't Veer P. Misreporting of energy and micronutrient intake estimated by food records and 24  hour recalls, control and adjustment methods in practice. The British journal of nutrition. 2009;101 Suppl 2:S73-85.
81.	Kirkpatrick SI, Midthune D, Dodd KW, Potischman N, Subar AF, Thompson FE. Reactivity and its association with body mass index across days on food checklists. J Acad Nutr Diet. 2012;112(1):110-8. Epub 2012/02/07.
82.	Dyer A, Elliott P, Chee D, Stamler J. Urinary biochemical markers of dietary intake in the INTERSALT study. The American journal of clinical nutrition. 1997;65(4 Suppl):1246S-53S.
83.	Börnhorst C, Huybrechts I, Ahrens W, Eiben G, Michels N, Pala V, et al. Prevalence and determinants of misreporting among European children in proxy-reported 24 h dietary recalls. Brit J Nutr. 2013;109(7):1257-65.
